# FLInt 2.0: robust and customizable single-shot integration in *C. elegans*

**DOI:** 10.1093/g3journal/jkag138

**Published:** 2026-05-28

**Authors:** Nawaphat Malaiwong, Porhathai Malaiwong, Chloe Kim, Michael O’Donnell

**Affiliations:** Department of Molecular, Cellular, and Developmental Biology, Yale University, New Haven, CT 06511, United States; Department of Molecular, Cellular, and Developmental Biology, Yale University, New Haven, CT 06511, United States; Department of Molecular, Cellular, and Developmental Biology, Yale University, New Haven, CT 06511, United States; Department of Molecular, Cellular, and Developmental Biology, Yale University, New Haven, CT 06511, United States

**Keywords:** *Caenorhabditis elegans*, transgenesis, safe landing sites, CRISPR, multiplex gene expression, WormBase

## Abstract

Transgenesis in *Caenorhabditis elegans* has revolutionized biological research by enabling the precise control of expression of both endogenous and exogenous genes. FLInt (Fluorescent Landmark Interference) was developed to integrate transgenes via CRISPR–Cas9 using visible changes in existing fluorescent protein expression strains. While the original FLInt method (FLInt 1.0) enabled a simple visual readout of potential transgene integration, the process was prone to false positives, leading to burdensome screening efforts. Here, we present an alternative FLInt strategy, FLInt 2.0, that reduces false positives by targeted CRISPR–Cas9 cutting of fluorescent protein landing sites in a manner which largely retains fluorescence in nonintegrative repair events but eliminates expression upon transgene integration. We demonstrate that this targeted approach maintains effective integration while significantly decreasing the proportion of false positives. Molecular and transmission analyses confirm that nonfluorescent F2 animals more reliably represent stably integrated multicopy transgenic lines. We show that integration efficiency and array transmission are influenced by DNA structure and composition, with linear DNA substrates promoting more robust array formation and insertion. We further show that multicopy transgene lines can be tailored to desired expression levels using a simple subsequent Cas9 targeting approach, reducing labor-intensive screening and increasing experimental throughput. Our strategy provides a robust, visually guided refinement of FLInt, offering a generalizable framework for improving site-specific transgene integration in *C. elegans*.

## Introduction

Integration of exogenous transgenes advanced the utility of *Caenorhabditis elegans* as a model organism by enabling genetic manipulation, protein localization studies, and targeted modulation of cellular processes (Nance and Frøkjær-Jensen 2019). Transgenes are typically introduced via gonadal microinjection of plasmid DNA, which assembles into large extrachromosomal arrays through de novo recombination (Stinchcomb et al. 1985; Mello et al. 1991). While these arrays allow robust expression—including of heterologous genes—they are not chromosomally integrated, resulting in unstable inheritance and mosaicism. To address this, a variety of integration techniques have been developed to ensure stable transmission and more uniform expression. Integration strategies vary in terms of copy number, site specificity, and procedural complexity. Single-copy insertion methods via transposon elements, e.g. MosSCI and MiniMos (Frøkjaer-Jensen et al. 2008; Frøkjær-Jensen et al. 2014), FLP recombinase (Nonet 2020, 2023), PhiC31 integrase (Yang et al. 2022), and CRISPR–Cas9 (Norris et al. 2015; Philip et al. 2019; Silva-García et al. 2019; Stevenson et al. 2020), offer precise targeting and reproducible outcomes. However, in many cases, multicopy transgene insertion is preferable due to increased expression levels or to combinatorially express multiple transgenes (Nance and Frøkjær-Jensen 2019).

Multicopy integration can be achieved in both targeted and random manners using CRISPR (Yoshina et al. 2016; El Mouridi et al. 2022; Yoshina and Mitani 2022; Malaiwong et al. 2023; Yanagi and Lehrbach 2024), UV or gamma irradiation (Mello and Fire 1995; Evans 2006; Mariol et al. 2013), PhiC31 integrase (Rich et al. 2025), and miniSOG (Noma and Jin 2018), respectively. Random insertion approaches via UV and miniSOG carry risks of off-target effects and often require labor-intensive outcrossing and/or mapping. Among targeted approaches, significant effort is often spent isolating rare integration events from a large population of false positives, as extrachromosomal array formation occurs at a higher frequency than genomic integration (Malaiwong et al. 2023). Recently established strategies to enrich for integrants include *unc-119* phenotypic rescue (El Mouridi et al. 2022; Yanagi and Lehrbach 2024), drug resistance (Dickinson et al. 2015; Stevenson et al. 2023; Rich et al. 2025), and fluorescent protein expression (Malaiwong et al. 2023). With the exception of the recently developed PhiAT approach (Rich et al. 2025), most of these methods require either cloning of transgenes into specialized targeting vectors or may yield false positives requiring extensive screening, which may serve as a barrier for uptake of these approaches for some of the *C. elegans* research community. FLInt (Fluorescent Landmark Interference), hereafter referred to as “FLInt 1.0,” was originally developed as a simplified multicopy integration approach that leverages CRISPR–Cas9-mediated excision of a defined locus bearing a fluorescent marker—a “safe harbor” site—and required no specialized reagents (Malaiwong et al. 2023). Many strains bearing these safe harbor sites exist (Frøkjær-Jensen et al. 2014) and by targeting the encoded tdTomato::H2B (Supplementary File S1) using a crRNA, FLInt enables detection of CRISPR activation in F1 offspring of injected animals and integrated transgenes in the F2 generation through loss of red fluorescence. However, despite its simple 1-shot injection protocol, FLInt 1.0 still demands substantial effort to identify true integrants due to numerous false positives arising from nonintegrative mutagenic repair events. This rate-limiting step underscores the ongoing tradeoff between procedural simplicity and the screening workload required for accurate identification.

Here, we present an improved single-shot approach for the generation of multicopy integrated transgenes which we have termed “FLInt 2.0.” FLInt 2.0 enables a simple visual method to distinguish true integrants from most mutagenic nonintegrants. We show that by introducing double-strand breaks (DSBs) via CRISPR–Cas9 in a landing site near the junction of the fluorescent protein coding domain and a 3′ UTR, many nonintegrative mutagenic events do not interfere with fluorescence. In contrast, array DNA integration disrupts this junction and results in complete elimination of fluorescent protein expression. As a result, fluorescent animals can be readily excluded during screening. We show that this approach simplifies identification of true integrants while requiring a screening effort that is comparable to typical extrachromosomal array generation methods. We further demonstrate that the expression level of integrated multicopy arrays can be customized through a subsequent round of CRISPR-mediated excision targeting sequences common to most cloning vectors. Together, this flexible, low-cost and streamlined method facilitates generation and modulation of integrated transgenes that should be accessible to most laboratories that are adept at microinjections.

## Materials and methods

### C. *elegans* culture

*C*. *elegans* strains were maintained using standard procedures (Stiernagle 2006). Wild-type (N2) and landing site integration strains were obtained from the *Caenorhabditis* Genetics Center: EG7837 (*oxTi712* [*eft-3*p::tdTomato::H2B::*unc-54* 3'UTR + *Cbr*-*unc-119*(+)] I; *unc-119*(*ed3*) III), EG7944 (*unc-119*(*ed3*) III; *oxTi553* [*eft-3*p::tdTomato::H2B::*unc-54* 3′UTR + *Cbr*-*unc-119*(+)] V), and were cultured at 16 °C or 20 °C prior to microinjection. Following injection, animals were maintained at 25 °C to accelerate developmental rates. As noted by CGC, tdTomato-expressing strains often appear sick when incubated at 25 °C, likely due to the ubiquitous expression of the fluorescent marker.

### crRNA designs

crRNAs for CRISPR–Cas9 were designed using the “Design CRISPR Guides” tool on Benchling and evaluated for efficiency and specificity with the IDT CRISPR–Cas9 guide RNA checker tool. The tdTomato-targeting crRNA (5′-AGATGGTCTTGAACTCCACC-3′) was modified from the original report to eliminate potential targeting of other red fluorescent protein coding sequences (Malaiwong et al. 2023), with additional guides designed against the tdTomato linker (5′-CTCCTCCGAGGACAACAACA-3′), the H2B::3′UTR junction (5′-ATGCTAACTAGTTTACTTGC-3′), the *C. briggsae unc-119* CDS::3′UTR junction (5′-TTATGCATCATATGAGTAGT-3′), and the ampicillin resistance gene (5′-ATTAATAGACTGGATGGAGG-3′). All crRNAs were verified to lack significant complementarity to the *C. elegans* genome or other fluorescent reporters, minimizing the risk of off-target effects.

### Molecular techniques

DNA fragments were amplified from plasmid templates using Phusion High-Fidelity PCR Master Mix with HF Buffer (NEB, #M0531) or KOD Hot Start DNA Polymerase (Sigma-Aldrich, #71086-3). Plasmids were constructed using the NEBuilder HiFi DNA Assembly Master Mix (NEB, #E2621), prior to transformation. Plasmids were extracted from bacteria cultures using Zyppy Plasmid Miniprep Kit (ZymoResearch, #D4020). Genotyping PCR was performed using OneTaq 2X Master Mix with Standard Buffer (NEB, #M0482L). The *unc-119(+)* rescue plasmid (pMOD351) was generated by PCR amplification of the *unc-119* sequence from the tdTomato landing strain EG7944 using primers listed in Supplementary Table 3 and cloned into the pMini2.0 vector (NEB, #E1202). To prevent CRISPR targeting of the *unc-119(+)* plasmid with crRNA(*unc-119*), mutations disrupting the guide sequence were introduced by site-directed mutagenesis (Supplementary Table 3).

### FLInt 2.0 ribonucleoprotein preparation

FLInt 2.0 ribonucleoprotein (RNP) complexes were assembled by combining 1 µL of 100 µM crRNA (IDT) targeting the desired region, 0.5 µL of 200 µM tracrRNA (IDT), 1 µL of 62 µM Cas9 endonuclease (IDT), and nuclease-free water to a final volume of 10 µL. This yielded final concentrations of 10 µM crRNA, 10 µM tracrRNA, and 6.2 µM Cas9. RNP complexes were incubated at 37 °C for 30 min prior to use. Aliquots were stored at −20 °C until injection. RNA sequences are listed in Supplementary Table 3.

### Injection mix preparation

Injection mixes were prepared using standard approaches (Supplementary Table 1). The plasmid of interest and co-injection marker were combined with 1 kb Plus DNA ladder (Invitrogen/ThermoFisher, #10787018) as carrier DNA to achieve a total DNA concentration of 100 ng/µL, except where indicated. Nuclease-free water was added to reach 8 µL, followed by addition of 2 µL FLInt 2.0 RNP mix to obtain a final volume of 10 µL. Prior to injection, the mixture was centrifuged at 12,000 rpm for 5 to 10 min to remove particulates. Injection mixes are detailed in Supplementary Table 1.

### Microinjection

Young adult hermaphrodites bearing the tdTomato::H2B landing site were maintained at 16 to 20 °C prior to injection. Well-fed young adults were selected using a paintbrush (Gibney et al. 2023), mounted on 2% dried agarose pads, and injected in one or both gonad arms following standard procedures (Evans 2006). After injection, 10 to 15 P0 worms were transferred together onto 6-cm NGM plates seeded with OP50 and incubated at 25 °C to promote array formation and subsequent integration events. For calculation of efficiency and accuracy data presented in Supplementary Table 2 and Fig. 2e and f, all injections, regardless of injection quality were included. In typical experimental pipelines, however, microinjections were repeated when low-quality injections were performed as indicated by the absence of any of the following: (i) highly variable expression of tdTomato in F1 offspring, indicating efficient CRISPR–Cas9 activity, (ii) many (50+) co-injection marker-positive F1 offspring, indicating efficient array formation, or (iii) many visible array-positive, tdTomato-negative worms in the F2 generation, indicating potential integrant candidates.

### Hygromycin treatment

Hygromycin stock solution (5 mg/mL) was prepared by diluting Hygromycin B (50 mg/mL) (ThermoFisher Scientific, #10687010) with milliQ water and stored at 4 °C. On day 3 postinjection, 500 µL of the stock solution was uniformly applied onto the 6 cm (10 mL) plate and bench dried for 20 to 30 min. As hygromycin is harmful, proper PPE should be applied during this step.

### Visual screening of candidates

Candidates for array integration were typically identified ∼6 d postinjection. The plates containing F2 progeny were screened under a fluorescence stereomicroscope, and candidate integrants were selected based on complete loss of tdTomato::H2B fluorescence. At this stage, dim residual tdTomato signal in F2 animals can increase false-positive selection; therefore, worms exhibiting partial or dim red fluorescence were avoided. In most experiments, candidates were initially selected solely based on loss of tdTomato::H2B without considering array expression—indicated by pharyngeal *myo-2*p::GFP (Vázquez-Manrique et al. 2010)—and tdTomato-negative animals were frequently observed to retain array expression. Selected animals were transferred to small (3.5 cm) NGM/OP50 plates for F3 screening to identify homozygous integrants using 100% transmission of array marker expression as a visual confirmation (Supplementary Table 2).

### Array integration via *unc-119* rescue selection

For enrichment via *unc-119(+)* positive selection, integrations were performed in the NJL4903 strain (nicTi600 [eft-3p::tdTomato::H2B::unc-119(-) *oxTi556] I; unc-119(kst33) III), which inactivates the *C.br unc-119* rescue allele and exhibits a temperature-sensitive *unc* phenotype at 25 °C—a generous gift from Nicolas Lehrbach and Christian Frøkjær-Jensen (Yanagi and Lehrbach 2024, Aljohani and Frøkjær-Jensen, unpublished reagent). All injection mixes are listed in Supplementary Table 2. Animals were maintained at a permissive temperature (16 °C) prior to injection and shifted to 25 °C postinjection to impose selection pressure. At 25 °C, nontransgenic progeny displayed impaired locomotion and reduced brood size, whereas animals carrying extrachromosomal arrays expressing *unc-119*(+) exhibited normal movement and apparent increased relative frequency in the F2 stage. Plates were screened after 6 d postinjection or after food exhaustion using standard FLInt 2.0 criteria: candidate integrants were identified by loss of tdTomato fluorescence in combination with co-injection marker expression and wild-type locomotion.

### F1 enrichment by manual transfer of array-positive animals

To provide a drug-free enrichment strategy that does not require hygromycin treatment or *unc-119* selection, 3 d postinjection, many (∼100) transgenic F1 progeny exhibiting robust co-injection marker expression were manually transferred together to 6 cm NGM/OP50 plates. This step effectively increased the proportion of array-bearing progeny entering the F2 generation while reducing background from nontransgenic siblings and preventing plate overcrowding. F2 integrant candidates derived from these enriched F1 plates were subsequently screened using standard FLInt criteria for loss of tdTomato::H2B in the presence of the array marker.

### FLInt-mediated integration of PCR fragments

For integration of overlapping PCR fragments, linear DNA was amplified by PCR bearing ∼20-nucleotide overlaps at adjacent junctions to facilitate in vivo recombination-based assembly within the germline (Kemp et al. 2007). Primers and PCR products are listed in Supplementary Table 3 and File S2. These overlapping fragments were co-injected with a plasmid-based fluorescent co-injection marker and a hygromycin selectable marker as listed in Supplementary Table 2. Stable lines were established and validated by genomic PCR across fragment junctions and Sanger sequencing to confirm correct in vivo assembly and integration at the landing locus. The prevalence of unintended recombination via random junction formation was evaluated in parallel by generating arrays via co-injection of a portion of the *rab-3* promoter along with cDNAs encoding mScarlet-I3, mTagBFP2, and GFP-C1, each bearing 20 bp overlaps with the *rab-3* promoter 3′ sequence. As a control, the same injection was performed with elimination of the 20 bp homology from GFP-C1. No *rab-3*p::GFP-C1 fusion events were detected in the latter case (Supplementary Fig. 4).

### Assessment of single-stranded oligonucleotide–mediated stop codon insertion

Single-stranded oligonucleotides (ssODN) were supplied as homology-directed repair (HDR) donors to introduce a synthetic stop codon immediately 5′ and 3′ of the crRNA(H2B) cut site (Supplementary Table 3 and Fig. 2). To determine ssODN insertion frequency via HDR, individual array-positive F1 progeny were lysed and used as PCR templates. Left and right HDR junctions were amplified separately using primers flanking the cut site and primers specific to the inserted sequence (Supplementary Table 3). PCR reactions were performed using OneTaq 2x Master Mix (NEB #M0482). Expected amplicons (∼0.4 kb for the left junction and ∼0.6 kb for the right junction) were resolved by agarose gel electrophoresis and visualized by gel imaging. The insertion frequency was calculated as the number of *myo-*2p::GFP-positive F1 animals showing the expected insertion divided by the total number of F1 animals analyzed ×100.

### Quantification of transgene copy number

Genomic DNA was prepared from *C. elegans* strains carrying integrated GFP constructs. For each sample, 10 adult worms were lysed in 20 µL worm lysis buffer (50 mM KCl, 10 mM Tris–HCl pH 8.3, 2.5 mM MgCl_2_, 0.45% NP-40, 0.45% Tween-20, 0.01% gelatin, and 60 µg/mL proteinase K). Lysates were incubated at 65 °C for 1 h and heat-inactivated at 95 °C for 15 min, then stored at −20 °C. Quantitative PCR (qPCR) was performed using 2 µL of worm lysate, corresponding to the DNA content of a single worm, as template in a 10 µL reaction with iTaq Universal SYBR Green Supermix (Bio-Rad) on a Bio-Rad CFX Opus 96 Real-time PCR System. Primer pairs were designed to amplify GFP (Forward primer: 5′-ATGAGTAAAGGAGAAGAACTTTTCACTGGAGTTGTC-3′ and Reverse primer: 5′-CCTTCAAACTTGACTTCAGCACCTG-3′) and the single-copy endogenous *act-1* gene (Forward primer: 5′-TTGCCGCTCTTGTTGTAGACAA-3′ and Reverse primer: 5′-ATTGGGTACTTGAGGGTAAGGA-3′) which served as the reference locus. Thermal cycling was performed with an initial denaturation at 95 °C for 10 min, followed by 40 cycles of 95 °C for 15 s and 60 °C for 1 min. Ct values for each strain were taken as the median of 3 technical triplicates. Transgene copy number relative to *act-1* was estimated by the comparative Ct method assuming 100% PCR efficiency. For each strain, ΔCt_strain_ was calculated as Ct_GFP_−Ct_act−1_. ΔΔCt_strain_ was calculated as ΔCt_strain_–ΔCt_single-copy_, where ΔCt_single-copy_ represented the single-copy reference strain, respectively. Relative copy number was determined as 2^−ΔΔCt(strain)^. For estimation of copy number precision, a standard curve was generated using the single-copy GFP strain (MOY225) as an input. Strains with estimated copy number above 150 fell outside the linear range for this standard curve and were therefore considered estimates. Strains analyzed included 3 integration strategies: MOY258–MOY262 (FLInt 1.0), MOY263–MOY267 (crRNA(Linker)), and MOY226–MOY235 (crRNA(H2B)).

### Statistical analysis

All statistical analyses were performed using RStudio. Sample sizes and relevant statistical tests are detailed in figure legends. Raw data are included in Supplementary material.

### Microscopy

Fluorescence microscopy was performed using a Leica DMi8 inverted fluorescence microscope equipped with a K5 sCMOS camera. Images were acquired using LAS X software. Adult worms were mounted on 2% agarose pads containing 10 mM tetramisole for immobilization. Single-plane captures and Z-stack images were acquired and processed using the Leica THUNDER Imager system.

### Quantification of fluorescence

#### tdTomato

Fluorescence intensity in F1 progeny was quantified at the L4/young adult stage following injection with the FLInt mixture. Three days postinjection, array-positive F1 worms were mounted on 2% agar pads, immobilized with 10 mM tetramisole for imaging. Red fluorescence was captured using an HI PLAN 4×/0.1 air objective (Leica TxR filter cube 11525310, Leica LED3 illumination, 17% FIM power, 100 ms exposure). For representative images, an HC PL APO 20×/0.80 air objective was used. tdTomato intensity was measured in ImageJ by defining a polygonal ROI tracing the worm's outline. Background fluorescence was measured from an adjacent region outside the worm and subtracted from the ROI signal. Final fluorescence values (A.U.) were calculated as *Intensity(ROI) – Intensity(background)*. An average per-sample-group background signal was used for background subtraction. tdTomato intensities from P0, F1, and F2 animals generated using FLInt 1.0 and FLInt 2.0 were plotted and compared. To plot F1 fluorescence phenotypes as stacked bar plots, raw tdTomato fluorescence intensity values measured in F1 progeny were classified into 4 categories based on predefined intensity ranges: dark (110 to 116), low (116 to 150), moderate (150 to 200), and bright (200 to 280).

#### Pharyngeal GFP

To compare the pharyngeal markers in tuning transgene copy number strategy, the intensity of the pharyngeal marker *myo-2*p::GFP (A.U.) was quantified by imaging young adult transgenic worms mounted on glass slides using a HI PLAN 4×/0.1 air objective (Leica GFP filter cube 11525314, Leica LED3 illumination, 17% FIM power, 10 ms exposure). Green fluorescence was measured from the region of the pharynx using a custom ImageJ macro to first identify a threshold mask which was used to generate ROIs for quantification.

#### CAN cell mCherry

CAN::mCherry expression was quantified by imaging young adult transgenic worms mounted on glass slides using a HC PL APO 20×/0.80 air objective. Red fluorescence was manually measured from the CAN cell region using ImageJ. A circular region of interest (ROI) was drawn over the CAN cell to obtain the signal intensity.

### Next-generation sequencing

CRISPR-edited animals were produced by microinjecting a CRISPR mix targeting the H2B region of the tdTomato landing site together with a co-injection marker (3 ng/µL *myo-2*p::GFP) and 97 ng/µL DNA ladder (Invitrogen). Injected worms were maintained at 25 °C. At 36 h postinjection, array-positive L2–L3 larvae were identified by marker expression and lysed either individually or as pooled samples. At the adult stage, array-positive F1 progeny were selected and processed as pooled lysates (15 worms per tube) or as single-worm lysates. A subset of array-positive F1 animals was propagated to generate F2 progeny. Adult F2 animals retaining the array were collected either as pooled samples (3 independent lines, 5 worms per line) or as individual worms. All lysates were stored at −20 °C prior to PCR. NGS amplicons were generated using KOD Hot Start DNA Polymerase (Sigma-Aldrich, #71086-3) in 50 µL reactions containing 2 µL of worm lysate with locus-specific primers (Supplementary Table 3). Reactions were purified using the QIAquick PCR Purification Kit (QIAGEN, #28106) and processed by Quintara Biosciences via 250 bp paired-end Illumina sequencing.

### Analysis of NGS amplicon sequences

INDEL variants were extracted, quantified, and compared across nonedited controls F1 and F2 animals. Raw reads were filtered above Phred quality ≥30 using Fastp. Sequence depth was between 30K and 80K reads per sample after filtering. Filtered reads were then run through a custom script to count unique sequences. Initial observations using unedited worm sequence reads indicated PCR or sequencing errors occurred at a rate of <1%, so this was used as a cutoff for quantification of edited alleles. All alleles above this threshold were then aligned using a custom python script, and alignments were manually inspected to confirm that most sequence variants remaining occurred at or near the intended cut site.

## Results

### Specific CRISPR-mediated cutting of safe harbor sites preserve fluorescence in the absence of integration

**Fig. 1.**
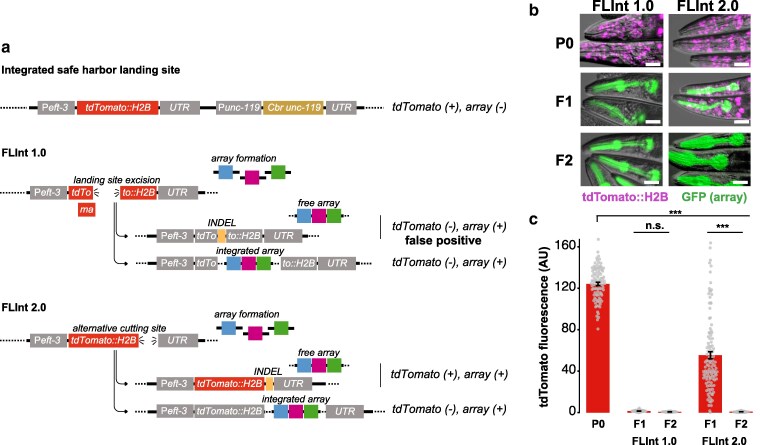
Revised FLInt enables phenotypic discrimination of true integrants. a) Schematic of the original (FLInt 1.0) and revised (FLInt 2.0) strategies at miniMos landing sites (*eft-3*p*::tdTomato::H2B::unc-54* 3′ UTR*; Cbr-unc-119*(+)). In FLInt 1.0, Cas9 cutting within the tdTomato coding region frequently generates INDEL alleles that abolish tdTomato expression, producing tdTomato-negative false positives. In FLInt 2.0, the cut site is shifted toward the 3′ region, allowing indel alleles to retain tdTomato expression, while array integration is predicted to disrupt tdTomato expression. b) Representative fluorescence images of P0, F1, and F2 animals from FLInt 1.0 and FLInt 2.0 showing GFP pharyngeal co-injection marker and tdTomato nuclei. Scale bar, 50 µm. c) Whole-animal tdTomato fluorescence intensity from animals of the indicated generation, quantified by measuring the mean red-channel signal using a segmented line ROI in ImageJ. *n* = 115 animals from a single injection for each condition. Dots are individual animals and bars are mean ± SEM. ns, not significant; ***P < 0.001; ANOVA with post hoc Tukey adjustment for a family of 5 comparisons.

Characterization of previously generated integrated transgenic worms using the FLInt 1.0 method indicated that there were principally 2 classes of animals lacking fluorescence after injection: those bearing small insertions or deletions (indels) and those containing large, multicopy array insertions (Malaiwong et al. 2023). The former class represented most isolated F1 worms, and are thus considered “false positives,” representing increased experimental effort and screening. Because these classes were initially indistinguishable, we reasoned that maintaining fluorescence in animals bearing small indels would improve selection accuracy by ensuring that only true positive integrants—which lose the tdTomato reporter through extrachromosomal array insertion—would become nonfluorescent only when homozygous in the F2 generation. To test this idea, we identified CRISPR target sites that are predicted to generate different fluorophore expression levels depending on the mechanism of gene editing, focusing on a collection of existing chromosomally integrated single-copy transgene strains that express ubiquitous nuclear-localized tdTomato (*eft-3*p::tdTomato::H2B::*unc-54* 3′ UTR; *Cbr*-*unc-119*(+)), enabling efficient visual screening. We targeted sites that are predicted to preserve an intact promoter–coding sequence–UTR configuration after the generation of small indels (Fig. 1a; Frøkjær-Jensen et al. 2014).

We first targeted the end of the H2B region, just before the 3′ UTR (crRNA(H2B) (Fig. 1a, Supplementary Table 3). Because CRISPR-induced DSBs in *C. elegans* are frequently repaired by nonhomologous end-joining, microhomology-mediated end-joining (Friedland et al. 2013), or synthesis-dependent strand annealing (Paix et al. 2017; Farboud et al. 2019), we expected that random small indels would preserve a functional portion of tdTomato::H2B and maintain a functional 3′ UTR, restoring mRNA integrity and fluorescence despite loss of a CRISPR–Cas9 cutting site. To reduce edits in the coding region or tdTomato::H2B, we designed our crRNA on the antisense strand, which is predicted to result in indels predominantly 5′ to the CRISPR cut site, which is 3′ relative to the tdTomato::H2B reading frame (Farboud et al. 2019). Unless otherwise indicated, for all injections, we included a *myo-2*p::GFP; *rps-0*p::hygR co-marker plasmid (pMOD346) to mark array-positive animals for visual and antibiotic selection (Supplementary Tables 1–3). Three days postinjection, we screened for F1 progeny showing pharyngeal GFP expression (indicative of array formation) and measured tdTomato fluorescence intensity (indicating potential presence of CRISPR-generated edits). Due to high efficiency of CRISPR–Cas9 relative to extrachromosomal array formation, we assumed that most array-positive (GFP+) worms bore chromosomal edits, which was supported by amplicon sequencing of pooled or single F1 GFP+ worms (Supplementary Table 4). Thus, we considered GFP+ F1 animals likely to be a representative population of CRISPR-induced end-joining repair events at a given crRNA cut site. Compared with F1 progeny from FLInt 1.0, indel-bearing animals edited via crRNA(H2B) exhibited higher, but variable tdTomato intensities, suggesting that either fluorescence is retained after CRISPR-mediated cutting at this site or that CRISPR targeting is incomplete (Fig. 1b and c, Supplementary Table 5). In contrast, F2 animals bearing chromosomally integrated plasmids appeared to completely lack tdTomato expression in both approaches (Fig. 1c, Supplementary Table 5).

To determine whether this difference in fluorescence via crRNA(H2B) may simplify the integrant selection process over FLInt 1.0, we compared the accuracy of candidate selection between integration via FLInt 1.0 method and via crRNA(H2B). In FLInt 1.0, candidate integrants were selected at the F1 stage and approximately 1 in 20 (∼5%) of those candidates contained true integration events (Malaiwong et al. 2023). To directly compare the selection process between these 2 methods, we selected array-positive (GFP+) F1 worms derived from pooled CRISPR-targeted P0 animals, reflecting independent events. We then compared the proportion F1 animals giving rise to approximately 75% array-positive offspring F2 (reflecting either high array transmission or potential integrants) and among these plates selected tdTomato-negative F2 worms (6–8 from each) to identify potential integrants in the F3 generation (Supplementary Tables 2 and 6). Integration events were identified when any of these F2 animals produced 100% array-positive F3 offspring. This analysis showed that while FLInt 1.0 has a higher integration efficiency compared with crRNA(H2B) based on the proportion of F1 offspring giving rise to integrants, the proportion of candidates at the F2 stage representing true positive integrants was higher for crRNA(H2B) (Supplementary Table 6).

We suspected that this difference in fluorescence—and apparent increase in candidate selection accuracy—would allow us to bypass the labor-intensive F1 selection process employed in FLInt 1.0, while simply pooling injected worms together for selection at the F2 stage. To facilitate candidate selection this stage, we injected a plasmid containing both a visible marker and hygromycin selectable cassette. Three days after injection, pooled F1 progeny were treated with hygromycin to select for animals expressing the HygR resistance gene in sufficient levels to enable survival. Six days postinjection, we screened F2 progeny for 2 key features: (i) complete loss of tdTomato fluorescence, and (ii) presence of GFP expression. Worms meeting both criteria were singled to individual NGM/OP50 plates. After incubation at 25 °C for 3 days, F3 progeny from each line were examined for 100% transmission of the array marker to confirm stable genomic integration. From F2 candidates injected with crRNA(H2B), ∼20% represented true positive integrants (*N* = 7 injections of 15 P0 each, *n* = 95 candidates, Supplementary Table 2). We were unable to identify successful integration events using the same pooling strategy with FLInt 1.0. Compared to the standard FLInt 1.0 approach, this represented a roughly 5-fold increase in candidate selection accuracy and required 10-fold fewer plates (Supplementary Table 2). We have termed this F2-based fluorescence selection process “FLInt 2.0.” While it is possible alternative culture or selection methods might improve FLInt 1.0 accuracy, these data suggest that the benefits of FLInt 2.0 over FLInt 1.0 lie in distinguishing true positive integrants from false positives via maintenance of fluorescence in the latter case via FLInt 2.0.

### An intact tdTomato::H2B and 3′ UTR is essential for maintenance of fluorescence in nonintegrated animals

**Fig. 2.**
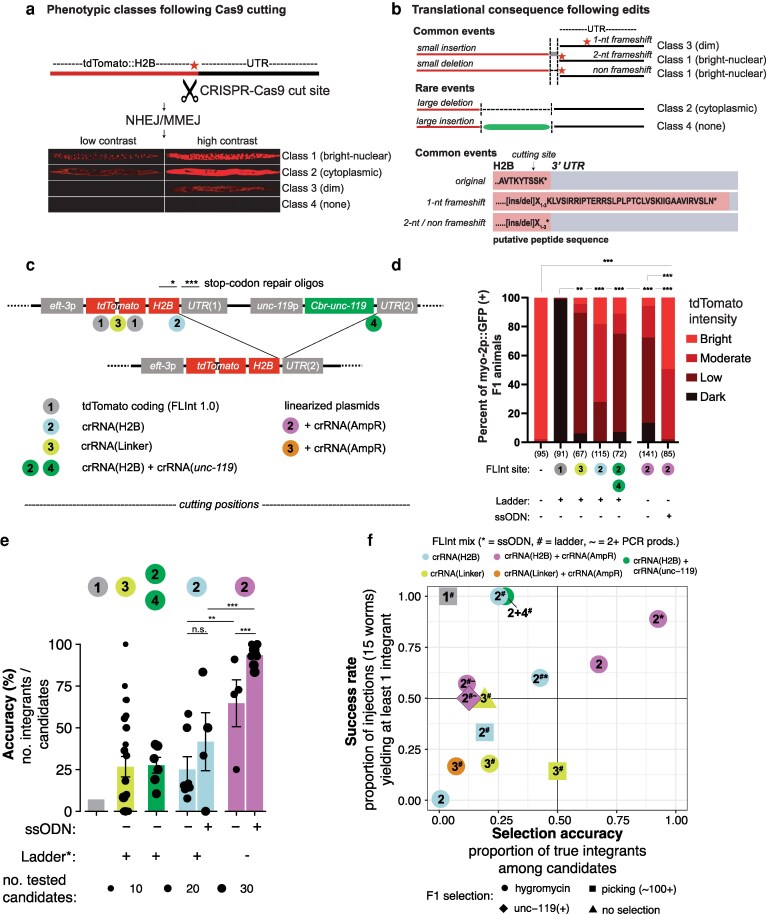
Cutting-site location determines tdTomato repair outcomes and integration accuracy. a) Schematic of tdTomato::H2B::3′ UTR showing the crRNA(H2B) Cas9 cut site and stop codon (star). Representative tdTomato phenotypes of isolated, isogenized progeny following repair are classified into 4 categories: class 1 (bright nuclear), class 2 (cytoplasmic), class 3 (dim), and class 4 (no signal), displayed at low (left) and high contrast (right). b) Predicted translational consequences of different repair outcomes. Top, small insertions or deletions (grey shading and dashed lines, respectively) generating either 2-nt frameshifts or nonframeshift alleles preserve bright, nuclear tdTomato expression via alternative stop codons (class 1 alleles). 1-nt frameshift alleles frequently result in dim tdTomato expression (class 3), whereas (middle) large insertions or deletions (grey dashed line or green oval, respectively) disrupt tdTomato localization or expression (classes 2 and 4). Bottom, putative peptide sequences generated from reading frame shifts as indicated. c) Schematic of alternative CRISPR cutting strategies at the tdTomato::H2B::3′ UTR–*Cbr-unc-119*:: 3′ UTR locus. Cutting sites include tdTomato coding sequence (site 1, FLInt 1.0, grey circles), H2B 3′ coding sequence (site 2, FLInt 2.0, blue and purple circles), tdTomato linker (site 3, yellow and orange circles), dual cuts at H2B 3′ CDS and *unc-119* 3′ CDS (sites 2 + 4, green circles). Injections including crRNA(AmpR) are shown with alternate colors to indicate the presence of linearized DNA. d) Proportions of tdTomato expression phenotypes (bright, moderate, low, dark) among F1 progeny selected by the *myo-2*p::GFP co-injection marker for each cutting strategy shown in (c). Note, these F1 categories are distinct from those of isogenic lines shown in (a). The number of F1 animals derived from a single injection batch is shown below plots. ***P < 0.001; **P < 0.01; ANOVA with post hoc Tukey adjustment for a family of 7 pairwise comparisons. e) Integration accuracy, defined as the proportion of selected candidates that were confirmed to be true integrants, among cutting strategies shown in (a). “+” and “−” below plots indicated the presence of and absence of ssODNs/ladder, respectively. Individual data points represent independent injections of 15 animals each; bars indicate mean ± SEM; dot size indicates the number of selected candidates. ***P < 0.001; **P < 0.01; n.s., not significant; binomial generalized linear model with Tukey post hoc adjustment for a family of 4 comparisons. f) Relationship between integration accuracy and success rate, defined as the proportion of successful injections for the cutting site categories shown in (a). Shape indicates F1 selection method. “*” indicates injections including ssODNs. “#” indicates injections including DNA ladder. “∼” indicates injections including multiple PCR products.

While maintenance of fluorescence expression in FLInt 2.0 reduced the fraction of false-positive-integrant animals (those bearing low/no tdTomato expression but without an integrated transgene), there nonetheless appeared to be several different classes of fluorophore expression phenotypes generated, potentially confounding the identification of true positive integrants. To better understand the molecular basis of indel-mediated maintenance of tdTomato expression, we examined the nature of repair among animals that did not carry integrated arrays. From injections using crRNA(H2B), we selected 4 classes of transgenic F2 animals based on their distinct tdTomato expression phenotypes: (i) bright nuclear tdTomato, (ii) cytoplasmic tdTomato, (iii) dim tdTomato, and (iv) non-tdTomato (Fig. 2a). Genomic DNA from isogenized lines derived from each category was extracted, and the CRISPR-targeted region encompassing the H2B 3′ UTR junction was amplified via PCR and sequenced (Fig. 2b). All sequenced animals in classes 1 and 3 carried edits at the intended cut site, confirming that CRISPR-mediated cutting is efficient among array-positive animals (Supplementary Fig. 1a–c). Edited class 1 alleles (high tdTomato expression, *n* = 6 analyzed) restored either an in-frame junction between H2B and the *unc-54* 3′ UTR or resulted in frameshift mutations followed almost immediately by an alternative stop codon in the 3′ UTR, consistent with functional recoupling that stabilizes mRNA and maintains nuclear fluorescence (class 1 alleles, Fig. 2, a and b, Supplementary Fig. 1a–c). A second, rare class of alleles (class 2, *n* = 7 analyzed, Fig. 2a and b, Supplementary Fig. 1c) of which only 2 were able to be definitively amplified using alternative, more distal PCR primers, appeared to bear larger deletions in the H2B coding sequence. Class 2 alleles exhibited bright, cytoplasmic tdTomato in edited worms. Both of these first 2 classes of alleles were expected and are easily distinguished from true positive integrants. However, the third class of tdTomato expression alleles (dim class 3, *n* = 9 analyzed, Fig. 2a and b) indicated the presence of frameshift mutations followed by long stretches of amino acids prior to a stop codon, potentially destabilizing the fluorescent protein (Supplementary Fig. 1a and b).

We hypothesized that these extended translation alleles could be eliminated by modifying the editing process via inclusion of single-stranded oligonucleotide repair templates (ssODN) intended to introduce stop codons in all 3 reading frames in addition to the existing stop codons in the 3′ UTR. The inclusion of ssODNs largely eliminated dimly fluorescent worms in F1 offspring of injected animals (Fig. 2d, Supplementary Table 2). To ascertain the mechanism of this ssODN-dependent change in tdTomato fluorescence, we performed genotyping PCR on FLInt 2.0 targeted F1 worms using primers complementary to the injected ssODN sequences. We found that precise HDR-mediated insertion of the repair oligos occurred only at modest frequency (∼10%) (Supplementary Table 7). With the caveat that F1 animals may not represent the spectrum of heritable editing events, this suggests that either introduction of HDR-oligo-dependent stop codons does not fully explain differences in F1 offspring fluorescence or that we may be missing partial oligo integration events. To distinguish between these possibilities, we performed Illumina amplicon sequencing of pooled *myo-2*p::GFP-positive F1 offspring from injected P0 animals either with or without ssODNs. Consistent with the PCR-based genotyping, we found that only a minority of sequenced alleles contained evidence of complete ssODN insertion (Supplementary Fig. 2a). Instead, while only truncated portions of the ssODN were typically inserted, this tended to result in predicted translational termination codons close to the insertion sites (Supplementary Fig. 2). HDR inclusion also reduced the occurrence of dim F2 phenotypes that complicate visual screening (data not shown). Consistently, inclusion of ssODNs increased F2 selection accuracy to ∼40% (*N* = 4 injections of 15 P0 each, *n* = 68 candidates, Supplementary Table 2). These results suggest that maintenance of fluorescence in nonintegrated array-positive worms reduces the number of false-positive integrants during pooled selection.

### Improvement of FLInt 2.0 selection accuracy

**Fig. 3.**
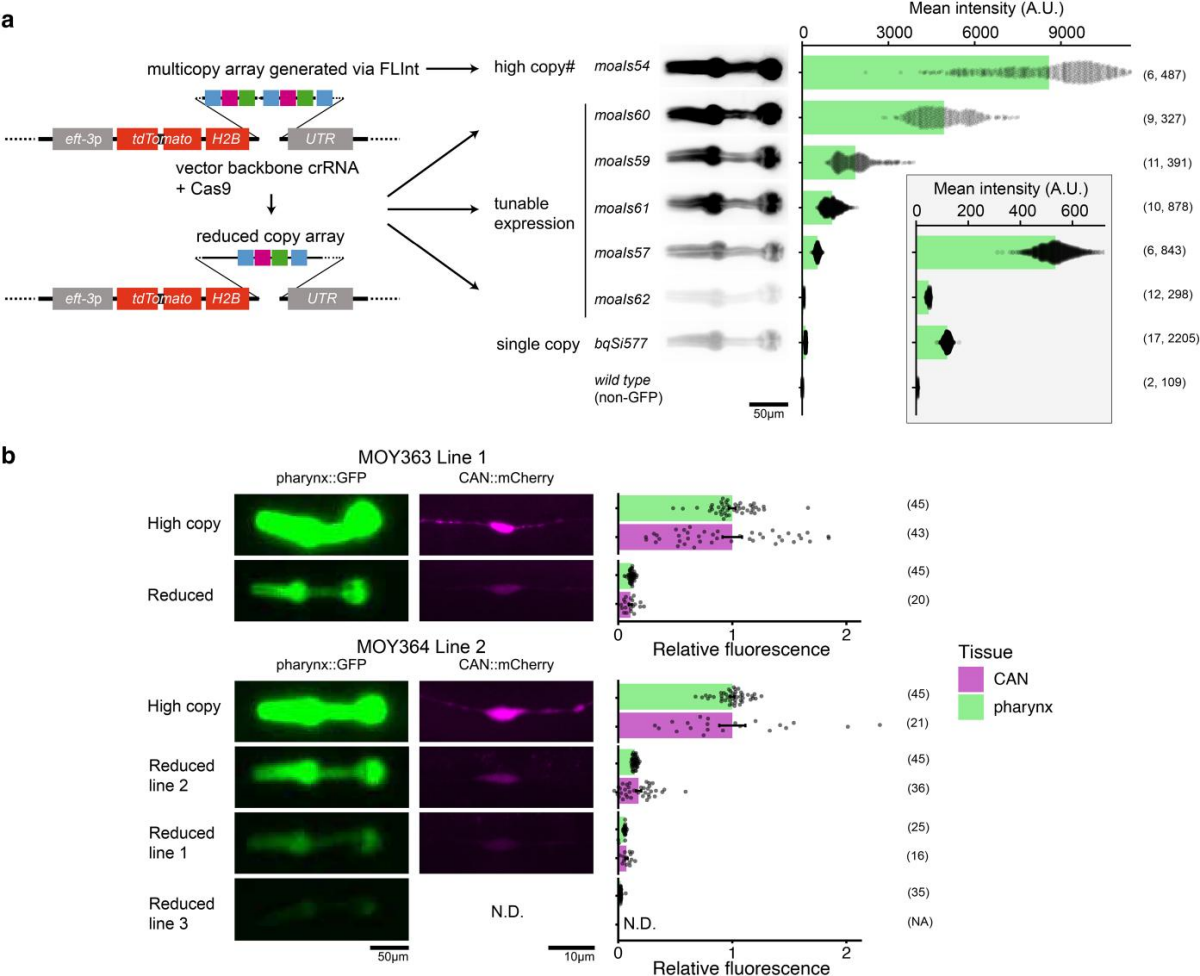
FLInt enables tunable transgene expression by reducing integrated array copy numbers. a) Left, schematic illustrating conversion of a high-copy number array generated via FLInt 2.0 into a reduced-copy lines after CRISPR–Cas9 cleavage of the vector backbone. Targeting the backbone decreases array copy number variably among progeny, resulting in tunable expression levels after selection. Middle, representative fluorescence images of independent lines with variable copy numbers derived from MOY301 *moals54* [*myo-*2p::GFP]: *moals60*, *moals59*, *moals61*, *moals57*, *moals62*. Strain *bqSi577* (single-copy *myo-*2p::GFP via MosSCI) is shown alongside wild-type controls. Right, quantification of pharyngeal GFP fluorescence intensity (A.U.). Each dot represents the average fluorescence of an ROI in an individual animal measured after isolation of isogenic lines. Scale bar, 50 µm. Inset shows detailed distribution of GFP expression among low copy lines. Number of images and worms analyzed per transgenic line, respectively, are shown on the right. b) Correlation of expression of pharyngeal GFP and CAN mCherry expression in high copy and reduced copy transgenic lines after targeting vector backbone sequence with crRNA(AmpR). Upper and lower panel represent copy number reduction lines derived from independent integrated multicopy arrays. Each dot represents the average fluorescence of an ROI in an individual animal divided by the population average expression for the respective tissue type in the multicopy parental strain. Bars indicate mean; error bars are SEM. Scale bars are indicated. N.D., not detectable. The number of worms analyzed per transgenic line is shown on the right.

Despite the increased selection accuracy achieved via FLInt 2.0, in our initial experiments, there remained, however, a significant proportion (∼60–80%) of class 4 (tdTomato-negative) isolates that did not appear to harbor stable plasmid integration events, as measured by 100% transmission of the *myo-2*p::GFP (Fig. 2e and f). We were unable to obtain PCR amplicons of the safe harbor locus from any of these isolates (*n* = 9), suggesting that these animals likely bore large, unintended insertions or deletions. We suspected that most of these false-positive-integrant animals contained extrachromosomal arrays. Complex extrachromosomal arrays—those containing a complement of heterogeneous DNA sequences—are known to be more stably expressed than simple homogeneous sequences (Kelly et al. 1997). However, de novo formation and inheritance of extrachromosomal arrays does not simply rely on DNA complexity per se, but rather an interaction between DNA AT-base composition and unknown other factors (Lin et al. 2021). We reasoned that elimination of carrier DNA might bias toward the inheritance of integrated arrays over inheritance and/or expression of extrachromosomal arrays. As expected, injections of low concentrations of *myo-2*p::*GFP* plasmid with and without carrier DNA both resulted in identification of GFP-positive F1 animals, but inheritance and/or expression beyond F1 required the co-injection of carrier DNA (Supplementary Table 8). We were unable to isolate apparent integrants via injection of circular plasmids without carrier DNA (*N* = 8 injections of 15 P0 each, *n* = 0 candidates, Supplementary Table 2 and Fig. 3). We note that we cannot distinguish between lack of transgene inheritance and transgene silencing in these experiments. The inability of circular plasmids to transmit effectively as extrachromosomal arrays was expected (Mello et al. 1991; Kelly et al. 1997), but the apparent absence of integration was not.

We considered the possibility that linearized plasmids may facilitate transgene integration via FLInt. To introduce linearized plasmid DNA, we co-injected a crRNA targeting the ampicillin resistance gene in common vector backbones (“crRNA(AmpR),” Supplementary Table 3). Linearized plasmids were capable of integration even in the absence of DNA ladder (Fig. 2e and f, Supplementary Table 2). This suggests that the presence of linear DNA—whether as carrier DNA or linearized plasmid—facilitates integration, potentially by promoting recombination or concatemer formation during array assembly. Elimination of carrier DNA and inclusion of ssODN donors further increased the true positive integrant identification accuracy of linearized plasmids to nearly 100% (Fig. 2e and f, Supplementary Table 2). Because the accuracy of integrant selection at this stage is much higher than FLInt 1.0, this corresponds to a dramatic reduction in the screening workload required to identify true integrants.

### Alternative crRNA sites and the estimation of integration efficiency

Mechanisms of DNA repair vary depending on the nature of the DSB as well as the types of donor DNA available for repair (Paix et al. 2017; Farboud et al. 2019). Because FLInt 1.0, which uses 2 cutting sites, appears have increased integration efficiency over FLInt 2.0 as a proportion of F1 transgenic offspring, we considered the possibility that larger deletions induced by multiple DSBs might alter the efficiency of integration of large, multicopy arrays. We designed crRNAs targeting different positions within the same background strain that are predicted to restore fluorescence in the absence of frameshift mutations after editing by linking tdTomato::H2B to a downstream 3′UTR encoded in the *C. briggsae unc-119+* rescue cassette (Fig. 2c). To prevent the generation of uncoordinated progeny due to CRISPR-mediated disruption of the endogenous *unc-119* locus, we included an *unc-119*(+) rescue plasmid (pMOD355) engineered to lack the CRISPR target sequence. Excision of this larger fragment resulted in a similar proportion of dimly fluorescent worms in the absence of integration, indicating that frameshift mutations are also likely responsible for this class of worms in this alternative configuration (Fig. 2d). Integration with coincident deletion of the *Cbr-unc119* coding sequence did not increase selection accuracy relative to the single crRNA(H2B), indicating that a single DSB is sufficient for accurate integration in these conditions (*N* = 6 injections of 15 P0 each, *n* = 122 candidates, Fig. 2e, Supplementary Table 2). In contrast, a crRNA targeting the linker domain between the 2 tdTomato dimers (crRNA(Linker)) resulted in a larger proportion of dim, but still visible tdTomato-expressing offspring (Fig. 2c and d). Taken together, these results suggest that targeted insertion to the 3′ CDS of a fluorescent protein largely eliminates frameshift-induced false-positive-integrant identification via FLInt and that recoupling to a functional 3′ UTR enhances screening accuracy.

While selection accuracy provides a useful guide for choosing crRNA sites via FLInt 2.0, it does not necessarily correlate with the overall success rate, as defined by the probability of identifying an integrant from a given injection batch. For example, despite generating a large number of dimly fluorescent F1 offspring using crRNA(Linker), the selection accuracy of true integrants using this configuration was similar to that obtained using crRNA(H2B) (Fig. 2e). This is likely because the dimly fluorescent offspring are possible, yet difficult, to distinguish from an absence of fluorescence in these worms. However, identification of integrant candidates (tdTomato-negative) using crRNA(Linker) required the injection of many more P0 worms per batch, possibly because HDR repair in *cis* (via the tandem repeat of tdTomato) is highly efficient and may compete with, or occur prior to, transgene integration (Fig. 2f, Supplementary Table 2). We compared the probability of integrant isolation for each of the crRNA cutting sites on a per-batch basis over many injections to estimate the efficiency of transgene insertion (Fig. 2f). This analysis shows that the crRNA(H2B) method using ssODNs is both the most accurate and most efficient, typically requiring only 15 injected worms to reliably identify integrants, while requiring the use of only 10–20 screening plates total (Supplementary Table 2). This workload is comparable to the generation of extrachromosomal arrays in our hands.

### Alternative selection approaches and DNA sources

To reduce reliance on hygromycin treatment, which can introduce physiological stress and potentially influence metabolic state in *C. elegans*, we evaluated a drug-free enrichment strategy based on *unc-119* rescue. Following the conceptual framework of Yanagi and Lehrbach (2024), we performed FLInt 2.0 integration in a temperature-sensitive *unc-119(kst33)* background (Aljohavi and Frøkjær-Jensen, unpublished reagent), which displays near wild-type behavior at low temperature (16 °C) but becomes uncoordinated at 25 °C; this configuration also simplifies strain handling relative to the *unc-119(ed3)* animals (N. Lehrbach, personal communication). After injection and temperature shift, progeny lacking extrachromosomal arrays exhibited impaired locomotion and reduced brood size, whereas array-bearing animals expressing *unc-119(+)* showed restored movement and brood expansion, enabling behavioral enrichment without antibiotic selection. Candidate integrants were subsequently identified using standard FLInt 2.0 criteria based on loss of tdTomato::H2B in combination with co-injection marker expression. Integrants were recovered at a somewhat lower frequency to those observed using hygromycin selection (*N* = 6 injections of 15 P0 each, *n* = 111 candidates, Supplementary Table 2). This outcome may reflect variability in the degree of behavioral rescue conferred by extrachromosomal *unc-119*(+) expression—which can depend on array composition, expression level, and mosaicism—or because these experiments involved the integration of several plasmids along with carrier DNA (Supplementary Table 2).

As an orthogonal approach without behavioral or drug selection, we also performed manual enrichment of array-bearing F1 progeny. GFP-positive F1 animals were identified using a pharyngeal *myo-2*p::GFP marker and pooled together to establish array-enriched subpopulations prior to F2 screening. Screening for loss of tdTomato::H2B yielded candidate integrants at an accuracy of ∼19%, comparable to selection-based approaches (*N* = 3 injections of 15 P0 each, *n* = 26 candidates, Supplementary Table 2). Although this strategy requires manual picking and yields somewhat more variable results, it preserves FLInt 2.0 candidate screening logic while avoiding both antibiotic exposure and mutant genetic backgrounds (Supplementary Table 2).

**Fig. 4.**
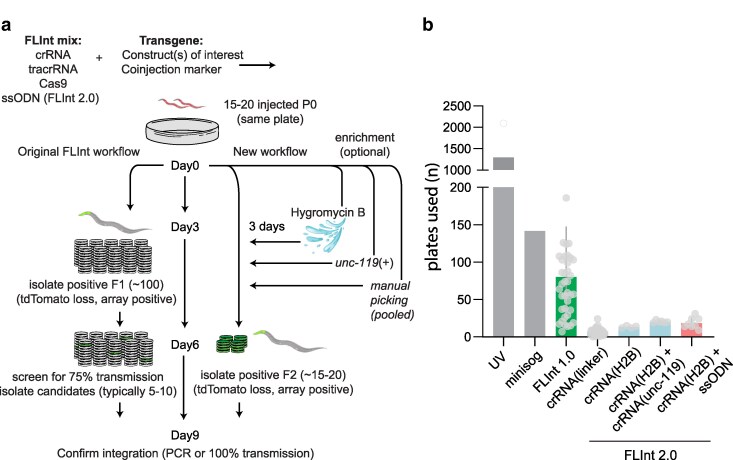
Comparison of FLInt workflows and plate usage across integration methods. a) Schematic outlining the original and updated FLInt workflows, showing the composition of the FLInt injection mix, the number of injected P0 animals, and the stages at which F1 and F2 candidates are isolated based on tdTomato loss and array positivity, followed by confirmation of integration by PCR or transmission analysis. b) Quantification of the number of plates used for isolating integrants across different integration strategies, including UV (Mariol et al. 2013), miniSOG (Noma and Jin 2018), FLInt 1.0 (Malaiwong et al. 2023), and FLInt 2.0 configurations as indicated. Each dot represents an independent experiment resulting from a single injection session; bars indicate mean plate usage.

Most of the efficiency and accuracy calculations we have shown involved injection of 1–2 plasmids (Supplementary Table 2). To test whether FLInt 2.0 can support integration of complex mixtures of input DNA, we performed injections comprising PCR products and either circular or linearized plasmid DNA (Supplementary Table 2). To facilitate screening for cointegration of multiple PCR products via in vivo recombination (Kemp et al. 2007), we used PCR fragments with overlapping homology (∼20 bp) intended to reconstitute either a promoter–cDNA junction, a cDNA-3′UTR junction, or both junctions, each resulting in the expression of distinct visible fluorescent markers, respectively (Supplementary File S2 and Table 3). We found that combinations of 2 or 3 PCR products in addition to multiple circular or linearized plasmids were reliably co-integrated using FLInt 2.0 screening combined with hygromycin selection (Supplementary Table 2 and Fig. 4). Together, these results indicate that FLInt 2.0 is compatible with multiple enrichment strategies and injected DNA compositions.

### Copy number control via backbone excision

Previously described approaches for transgene integration indicated that transgene copy number is often correlated with expression level, which is apparent when a transgene contains a fluorescent coexpression marker (Yoshina et al. 2016; Noma and Jin 2018; Malaiwong et al. 2023). Our previous experiments using FLInt 1.0 indicated that copy number correlated with the concentration of injected plasmids, but experimentally this required separate injections, increasing workload (Malaiwong et al. 2023). Preliminary examination of fluorescence levels in integrated transgenic lines generated using FLInt 2.0 indicated that, as in the case of FLInt 1.0, these likely reflected high-copy number transgenes. Consistent with this, qPCR analysis of GFP copy number in 20 independent lines derived from our different integration methods indicated that all contained >30 to 150+ copies, with no obvious effect of the cutting site on copy number (Supplementary Fig. 5).

While high-copy transgenes are preferable in many experimental contexts, it is often desirable to tailor copy number and expression level, which can be a laborious process using standard methods. We reasoned that we could selectively reduce transgene copy number simply via CRISPR-mediated excision. We used crRNA(AmpR) which is predicted to cut within the *beta-lactamase* (*bla*/*ampR)* gene contained in many common cloning vectors (Supplementary Table 3). Cas9 targeting of this site via crRNA injection in a high-copy number transgene line resulted in highly efficient generation of offspring with various levels of reduced expression of *myo-2*p::GFP (Fig. 3a, Supplementary Table 9). Some of these lines exhibited expression levels approximating that of a single-copy insertion (Fig. 3a). To determine whether *myo-2*p::GFP serves as a reliable proxy for the coexpression level of additional transgenes, we first generated multicopy integrated lines via FLInt 2.0 that coexpressed mCherry in the CAN cell, via the CAN-specific *ceh-23L*p (Wenick and Hobert 2004, Supplementary Table 3). Copy number reduction via crRNA(AmpR) injection in these lines leads to a consistent, relative reduction in expression of both GFP and mCherry, indicating that *myo-*2p::GFP reliably predicted the expression of array-encoded transgenes under these conditions (Fig. 3b). We caution that some plasmids have been reported to exhibit nonrandom distribution in integrated arrays (Rich et al. 2025), so these results may vary depending on the composition of arrays. These results indicate that high-copy-number transgenes generated via FLInt 2.0 can be easily tuned to desirable expression levels via excision using Cas9 and vector backbone targeting.

### Estimation of reagent and screening costs

An additional barrier to uptake of integration methods is the amount of effort, time, and resources required to identify desired strains. We therefore estimated the resources required to generate integrated strains in 2 different ways: hours of effort per integrant construct/line isolated and number of plates used. Based on reported efficiencies and in our experience, FLInt 1.0 and other comparable methods, including extrachromosomal array-based methods, all typically require the injection of ∼15 to 20 P0 animals per transgene and vary by experience, so we have excluded the injection time in our estimates (El Mouridi et al. 2022; Yoshina and Mitani 2022; Malaiwong et al. 2023; Yanagi and Lehrbach 2024). Because the selection process for FLInt 2.0 occurs in the F2 generation and animals are pooled after an injection session, this results in a significant reduction in time prior to screening, with only a few minutes required for addition of hygromycin 3 d after injection (Fig. 4a). While hygromycin treatment or alternative selection methods, such as *unc-119*(+) or manual F1 transfer can be omitted, this significantly reduced the screening time required to identify candidates in the subsequent F2 stage in our hands, by eliminating offspring from poorly injected animals or nontransgenic offspring. Identification and isolation of 15 to 20 coexpression marker-positive and tdTomato-negative F2 worms typically takes 30 min. Screening of F3 animals to distinguish between extrachromosomal array lines, heterozygous and homozygous integrant lines requires an additional 30 min. Because this approach usually resulted in the successful isolation of integrants, this equated to about 2 h of effort per integrated line in our hands.

Screening for integrant strains using conventional methods typically requires the isolation of many candidate lines, followed by selection or segregation analysis over multiple generations, resulting in the use of substantial quantities of materials (Fig. 4a and b). In contrast, FLInt 2.0 typically requires only about 15 to 20 plates in total prior to integrant identification (Fig. 4b). The cost of reagents (Cas9, crRNAs, oligos, plasmid, hygromycin) using this method is not prohibitive for most labs, when estimated per integrated line (Supplementary Table 10). Together, these results suggest that the combination of effort required and materials used in this method approximates that of stably inherited extrachromosomal array line generation in our experience.

## Discussion

Here, we describe a simple and accessible method for the generation of multicopy transgenes in *C. elegans.* Our method eliminates some existing barriers to the adoption of multicopy integrated transgenes over extrachromosomal arrays; this approach is affordable for most laboratories in terms of cost and effort and requires no specialized cloning or reagents other than Cas9 ribonucleoprotein complex and existing *C. elegans* landing site strains.

### Current approaches to generation of integrated transgenes

Recent advances in genome engineering have made the introduction of precise edits and insertions in *C. elegans* using CRISPR–Cas9 highly efficient (Dickinson and Goldstein 2016; Nance and Frøkjær-Jensen 2019). Similarly, the development of safe harbor landing sites in the genome via Mos1 (Frøkjaer-Jensen et al. 2008, 2012, 2014; El Mouridi et al. 2022) has enabled precise and efficient single-copy transgene insertion via FLP recombinase (Nonet 2020, 2023), CRISPR–Cas9 (Norris et al. 2015; Philip et al. 2019; Silva-García et al. 2019; Stevenson et al. 2020; El Mouridi et al. 2022) or PhiC31 integrase (Yang et al. 2022). In contrast, there are relatively few developed methods to efficiently integrate large multicopy or multiplasmid arrays in defined genomic loci, which may contribute to the continued use of extrachromosomal arrays in many labs when high levels of expression or the expression of multiple constructs is needed. *C. elegans’* natural capacity to readily generate complex, recombinant megabase-sized extrachromosomal arrays of foreign DNA with holocentric properties remains a unique strength among experimental organisms (Stinchcomb et al. 1985; Mello et al. 1991; Lin et al. 2021; Stevenson et al. 2023), yet inheritance of these arrays is meiotically and mitotically unstable and they are frequently silenced in the germline, limiting their robust experimental application.

Recently, El Mouridi et al. (2022) developed a method to integrate existing complex extrachromosomal arrays using CRISPR–Cas9-dependent excision and nonhomologous repair at safe harbor landing sites. These efforts have been complemented by approaches to integrate arrays in a single-shot injection, via integration at CRISPR–Cas9-generated DSBs in the *dpy-3* or *ben-1* genes (Yoshina and Mitani 2022), in safe harbor landing sites (Malaiwong et al. 2023; Yanagi and Lehrbach 2024) or via PhiC31 integrase-mediated integration (PhiAT) at defined loci (Rich et al. 2025). All of these methods have specific strengths; while some require multiple injection steps, cloning into specialized vectors or result in a nonoptimal proportion of false positives, methods such as PhiAT have very a high integration efficiency and the ability to generate many independent lines from a single injection that comes at a cost of a somewhat longer experimental isolation period than FLInt 2.0. In this work, we sought to develop methods that are experimentally simple, requiring only expertise that is comparable to extrachromosomal array construction. Ultimately, the choice of method depends on the strengths of the lab and the specific priorities of the experimental approach.

### Simplicity and generalizability of this system

The method we describe here requires no specialized cloning, is theoretically compatible with any plasmid or PCR product, and can be used to generate multicopy arrays from a single injection of ∼15 animals with very little screening effort and relatively few false positives. As with the FLInt 1.0 approach (Malaiwong et al. 2023), arrays generated using FLInt 2.0 typically contain high copy numbers. By co-injection with a readily visible or selectable transgene marker (e.g. *myo-2*p::*GFP*, *hygR*), screening time using this approach requires minutes, not hours, using a fluorescent stereomicroscope. We also show that copy number can be readily tailored to desired expression level using a simple additional CRISPR–Cas9 targeting of common sequences included in cloning vectors, likely eliminating the need for laborious injections of variable construct concentrations. A detailed protocol is actively maintained at the following URL: http://dx.doi.org/10.17504/protocols.io.eq2lyqm1pvx9/v3.

### Efficiency considerations

Under certain conditions, we were able to achieve nearly 100% selection accuracy using FLInt 2.0. We suspect, however, that similar selection accuracy may not be achievable when constructing highly complex arrays. Also, when measured as the proportion of F1 array-positive offspring harboring an integrated transgene, the integration efficiency of the most basic FLInt 2.0 implementation is lower than FLInt 1.0. While this may be due to either the multiple cutting sites in FLInt 1.0 or efficiency differences between crRNAs, in our experience, the benefits of reduced screening time in FLInt 2.0 clearly outweigh these differences in insertion frequency at the F1 stage. Moreover, although we cannot directly compare the efficiency of the optimized FLInt 2.0 to FLInt 1.0 and other methods given the alternative selection criteria, our anecdotal observations suggest that linearization, exclusion of carrier DNA and inclusion of ssODNs likely improves FLInt 2.0 efficiency. However, for laboratories in which the major limitation for transgenic line generation is injection quality, any reduction in efficiency may be critical. Under those conditions, we suggest using alternative 1-shot approaches such as PhiAT (Rich et al. 2025), FLInt 1.0 with modifications (Malaiwong et al. 2023; Yanagi and Lehrbach 2024), or methods to integrate existing arrays (El Mouridi et al. 2022). Ultimately, proper microinjection is a necessary step in any method to generate new, integrated transgenes in *C. elegans*.

### Pleiotropy and transgene selection

Our approach uses existing safe harbor sites which overexpress tdTomato as well as *C. briggsae unc-119*. Both elements may lead to pleiotropic effects in certain circumstances (Omi and Pujol 2021, N. Malaiwong, unpublished observations). Given that the FLInt 2.0 approach only requires repair events that link the existing tdTomato::H2B CDS with an existing 3′ UTR, there may be advantages to eliminating the existing *C. briggsae unc-119* cassette by using both crRNA(H2B) and crRNA(*unc-119*). Similarly, hygromycin B is toxic to humans, and high expression of the hygromycin resistance gene even in the absence of drug treatment may result in pleiotropic effects. For applications in which array composition is particularly important, we suggest opting for the somewhat more laborious manual selection method which does not require inclusion of selectable markers (Table 1).

**Table 1.** Comparison of enrichment strategies compatible with FLInt 2.0 integration.

Strategy	Strengths	Limitations	Recommended application
Hygromycin selection	High stringency enrichment; strong reduction of nontransgenic background	Antibiotic toxicity; may reduce brood size; requires drug handling	Large-scale integration experiments or high-throughput screens
Manual F1 enrichment	Drug-free; minimizes physiological stress	Labor-intensive; requires careful initial manual screening	Small-scale experiments or settings where drug selection is undesirable
*unc-119*(+) rescue selection	Genetic enrichment without antibiotics; temperature-dependent selection	Rescue efficiency may vary depending on allele strength and expression level	Laboratories preferring nonantibiotic selection strategies

### DNA composition impacts integration

Our observations further suggest that the structure and complexity of injected DNA influence both extrachromosomal array transmission and integration efficiency via FLInt 2.0. Linear DNA, whether provided as carrier DNA or linearized plasmids/PCR products, consistently promoted array inheritance and integration, whereas circular plasmids alone were inefficient. These findings support a model in which linear DNA facilitates concatemer formation and array complexity, thereby enhancing chromosomal integration (Mello et al. 1991). We also observed that inclusion of ssODNs increased selection accuracy and, anecdotally, integration efficiency. We suspect that this may be due to an unanticipated effect of homology of the injected ssODN with crRNA(H2B) (Supplementary Fig. 2). Previous reports have shown that spacer-complementary oligonucleotides inhibit CRISPR–Cas9-dependent nuclease activity (Han et al. 2025). If this is true in our experiments, why would this increase integration efficiency? We speculate that this apparently paradoxical observation may be related to the timing of extrachromosomal array formation. Extrachromosomal arrays form in the germline cytoplasm, typically hours after injection, while injected CRISPR–Cas9 nuclease, which bears a nuclear localization signal, may begin to cut genomic DNA on shorter timescales (Yuen et al. 2011; Nonet 2020). While the precise molecular mechanism remains unclear, the composition and topology of injected DNA appear to be key determinants of integration success (Table 1).

Finally, beyond conventional plasmid-based integration, we have seen that FLInt 2.0 is potentially compatible with integration of transgenes apparently assembled in vivo from overlapping PCR fragments (Supplementary Table 2 and Fig. 4). Although in vivo assembly has been previously described in the context of extrachromosomal arrays and in single-copy insertions (Kemp et al. 2007; Paix et al. 2016), its application to multicopy array formation followed by targeted chromosomal integration has not to our knowledge been previously demonstrated. While these experiments are preliminary and require further characterization to demonstrate the specificity of assembly, our results suggest that FLInt 2.0 can accommodate diverse DNA sources. However, we suspect that this property is likely shared among other multicopy insertion approaches as well. Importantly, independent verification of the composition of integrated arrays should be routinely performed regardless of the approach used.

### Future extensions

In our experiments, we have used FLInt 2.0 to generate relatively simple multicopy arrays (1–4 plasmids/constructs, with or without carrier DNA). While additional work is required to establish the upper limit for transgene complexity using this method, we anticipate that this should in theory approach that of highly complex extrachromosomal arrays analyzed in other work (Lin et al. 2021; El Mouridi et al. 2022; Stevenson et al. 2023). This suggests that integrated transgene library assembly should be readily tractable using this approach or combined with other existing approaches.

## Supplementary Material

jkag138_Supplementary_Data

## Data Availability

All referenced strains and plasmids are listed in Supplementary Table 3 and are available upon request. The authors affirm that all data necessary for confirming the conclusions of the article are present within the article, figures, and tables. Supplemental material available at G3 online.
