# Supplementary material for: FLInt 2.0: robust and customizable single-shot integration in C. elegans
Source: G3 (Bethesda). 2026 May 28;16(8):jkag138. doi: 10.1093/g3journal/jkag138 (PMC13439921; doi:10.1093/g3journal/jkag138)
Supplement: Supplementary file 1 [file jkag138_supplementary_data.zip › Figure_S1_G3-2026-406865.pdf]

Figure S1

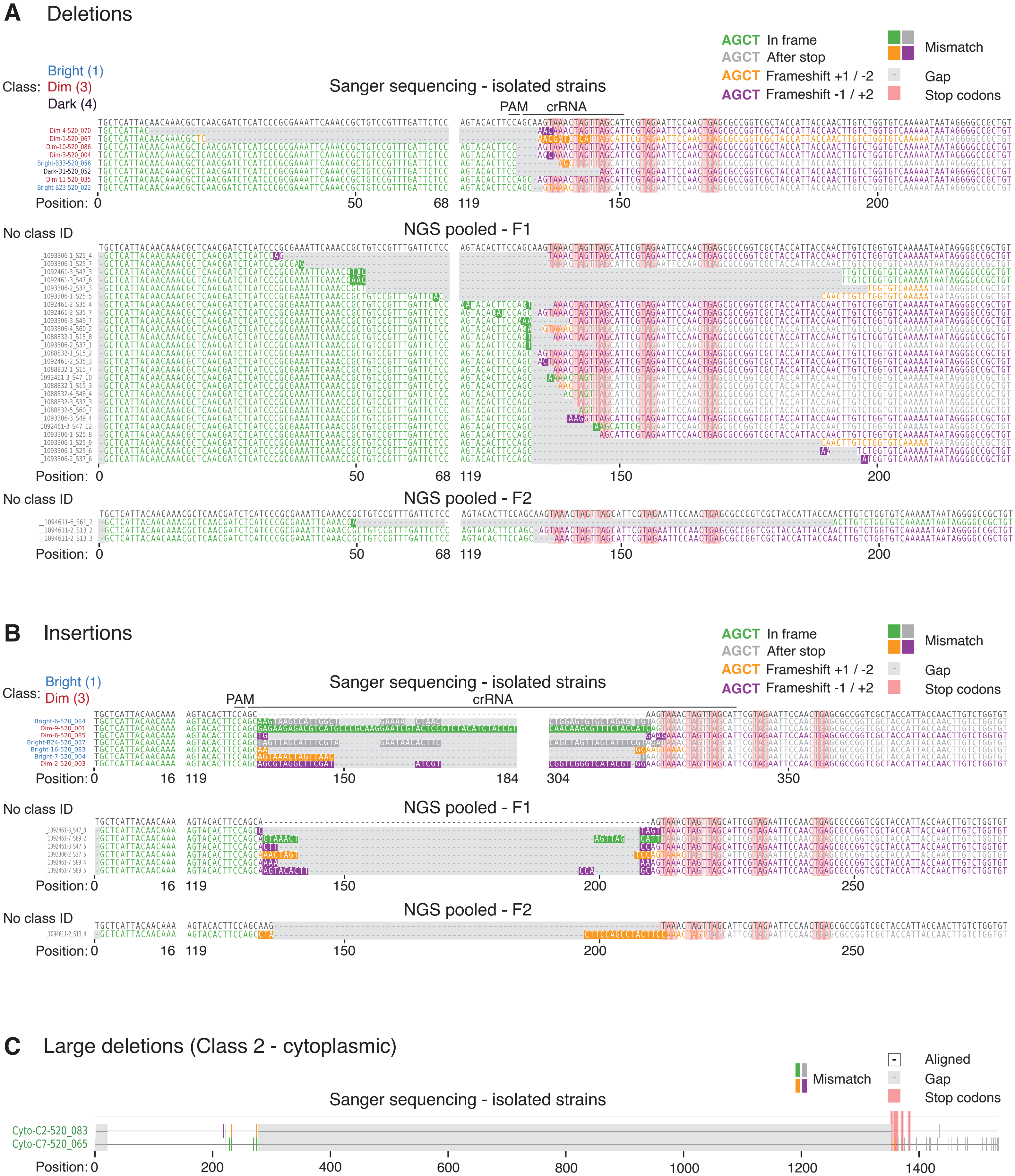
A Deletions
AGCT In frame
AGCT After stop
AGCT Frameshift +1 / -2
AGCT Frameshift -1 / +2
Mismatch
Gap
Stop codons
Class: Bright (1) Dim (3) Dark (4)
Sanger sequencing - isolated strains
PAM
crRNA
Position: 0 50 68 119 150 200
No class ID
NGS pooled - F1
Position: 0 50 68 119 150 200
No class ID
NGS pooled - F2
Position: 0 50 68 119 150 200
B Insertions
AGCT In frame
AGCT After stop
AGCT Frameshift +1 / -2
AGCT Frameshift -1 / +2
Mismatch
Gap
Stop codons
Class: Bright (1) Dim (3)
Sanger sequencing - isolated strains
PAM
crRNA
Position: 0 16 119 150 184 304 350
No class ID
NGS pooled - F1
Position: 0 16 119 150 200 250
No class ID
NGS pooled - F2
Position: 0 16 119 150 200 250
C Large deletions (Class 2 - cytoplasmic)
Mismatch
Aligned
Gap
Stop codons
Sanger sequencing - isolated strains
Cyto-C2-520_083
Cyto-C7-520_065
Position: 0 200 400 600 800 1000 1200 1400

Figure S1. Analysis of non-integrative genome edits derived from FLInt 2.0 injections.
(A-C) Multiple sequence alignments generated from either Sanger sequencing of isolated homozygous edited worms or Illumina amplicon sequencing of pooled offspring injected with crRNA(H2B), categorized by edit type (A) deletions, (B) insertions and (C) large deletions requiring extended PCR amplicons. (A-B) For each alignment, base coloring indicates the reading frame as follows: green, in-frame bases 5' to indel sites; grey, bases following a putative in-frame stop codon; orange, bases following a +1bp or -2bp frame-shift; purple, bases following a -1bp or +2bp frameshift. Preexisting termination codons are highlighted in red. Additional highlighted bases indicate mismatches from the reference, which are likely indels and colored coded in the same manner. For Sanger sequenced isolates, the left sequence ID is color coded according to the tdTomato expression class – indicated in parentheses: blue, 'bright' class 1 bright nuclear tdTomato; green, 'cytoplasmic' class 2 bright cytoplasmic tdTomato; red, 'dim' class 3 dim tdTomato; black, a single isolate was originally classified as 'dark' class 4 non-tdTomato, but upon closer examination was found to be very dim and likely class 3.
