# Supplementary material for: FLInt 2.0: robust and customizable single-shot integration in C. elegans
Source: G3 (Bethesda). 2026 May 28;16(8):jkag138. doi: 10.1093/g3journal/jkag138 (PMC13439921; doi:10.1093/g3journal/jkag138)
Supplement: Supplementary file 1 [file jkag138_supplementary_data.zip › Figure_S2_G3-2026-406865.pdf]

Figure S2

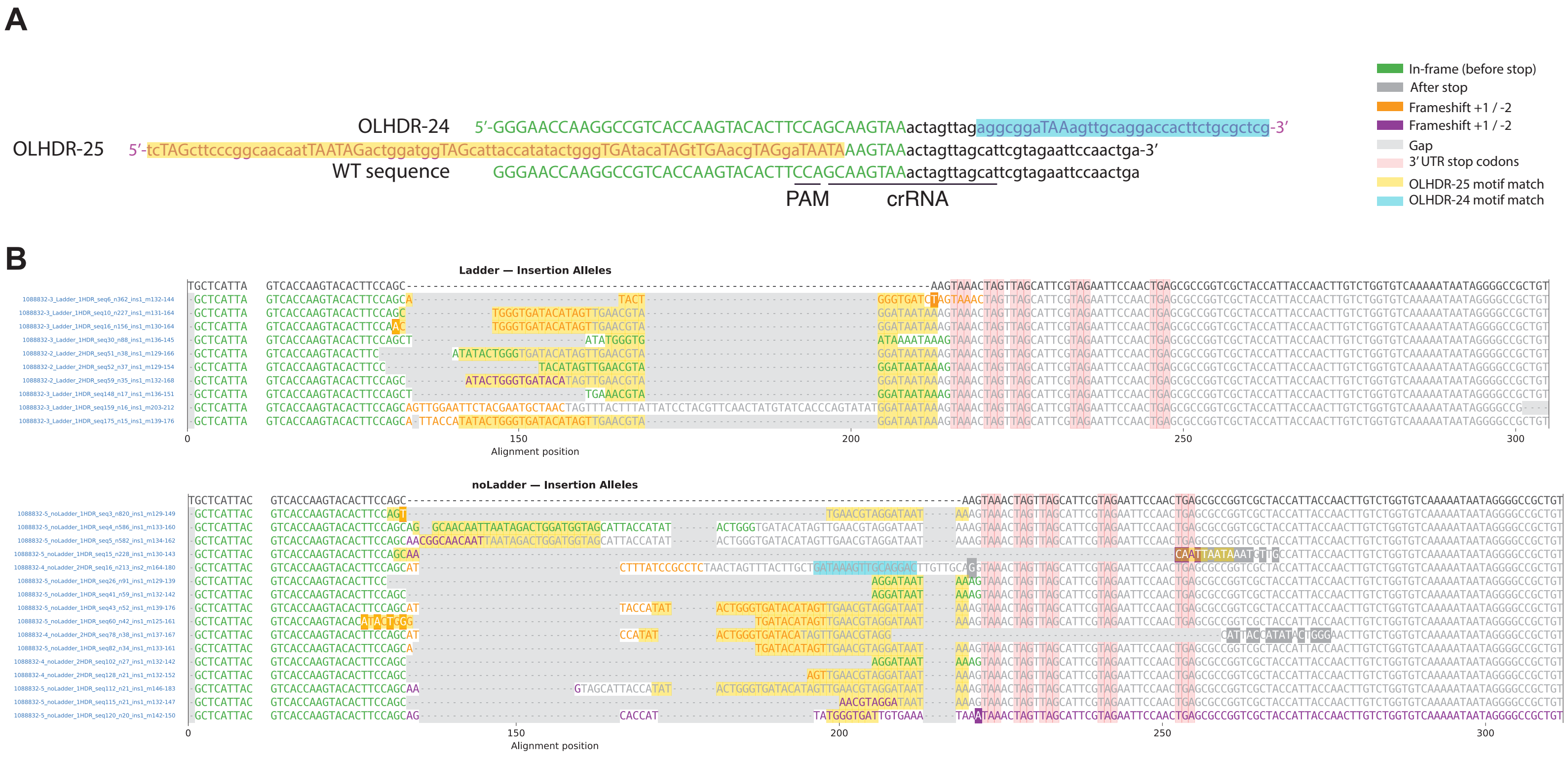

Figure S2. Effects of ssODNs on non-integrative repair via FLInt 2.0

(A) Alignment of ssODNs bearing multiple termination codons with sense strand of a tdTomato::H2B landing site. Position of crRNA(H2B) and corresponding PAM site on the antisense strand are indicated with horizontal lines below sequence. (B) Multiple sequence alignments generated from Illumina amplicon sequencing of pooled offspring injected with crRNA(H2B) along with ssODNs indicated in (A) with (top) and without (bottom) DNA ladder. For each alignment, base coloring indicates the reading frame as follows: green, in-frame bases 5' to indel sites; grey, bases following a putative in-frame stop codon; orange, bases following a +1bp or -2bp frameshift; purple, bases following a -1bp or +2bp frameshift. Preexisting termination codons are highlighted in red. Additional highlighted bases indicate putative inserted sequences derived from ssODNs OLHDR-24 (blue) and OLHDR-25 (yellow).
