# Supplementary material for: FLInt 2.0: robust and customizable single-shot integration in C. elegans
Source: G3 (Bethesda). 2026 May 28;16(8):jkag138. doi: 10.1093/g3journal/jkag138 (PMC13439921; doi:10.1093/g3journal/jkag138)
Supplement: Supplementary file 1 [file jkag138_supplementary_data.zip › Figure_S3_G3-2026-406865.pdf]

Figure S3

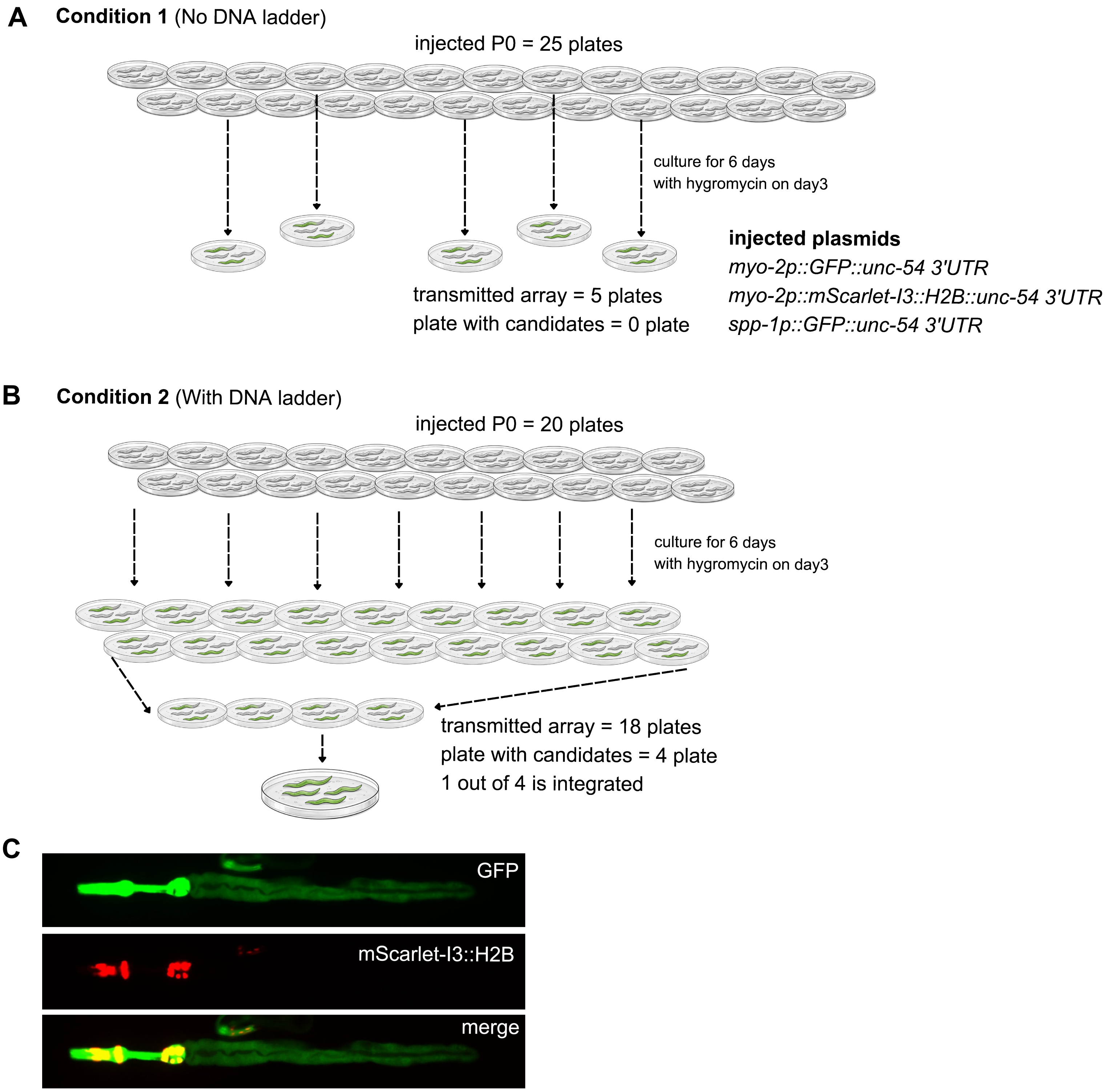

Figure S3. Effect of DNA ladder on transmission and candidate recovery during FLInt-mediated integration. (A) Experimental workflow under Condition 1 (no DNA ladder). A total of 25 P0 animals were injected and cultured for 6 days with hygromycin selection initiated on day 3. Five plates transmitted extrachromosomal arrays; however, no candidate integration plates were recovered. Injected plasmids included the 3 fluorescent markers in pharyngeal muscle and gut. (B) Experimental workflow under Condition 2 (with DNA ladder). Twenty P0 animals were injected and cultured under the same selection regime. Eighteen plates transmitted arrays, four candidate plates were identified and 1 out of 4 plate is integrated. (C) Representative fluorescence images of candidate animals showing GFP, mScarlet-I3, and merged channels.
