# Supplementary material for: FLInt 2.0: robust and customizable single-shot integration in C. elegans
Source: G3 (Bethesda). 2026 May 28;16(8):jkag138. doi: 10.1093/g3journal/jkag138 (PMC13439921; doi:10.1093/g3journal/jkag138)
Supplement: Supplementary file 1 [file jkag138_supplementary_data.zip › Figure_S4_G3-2026-406865.pdf]

Figure S4

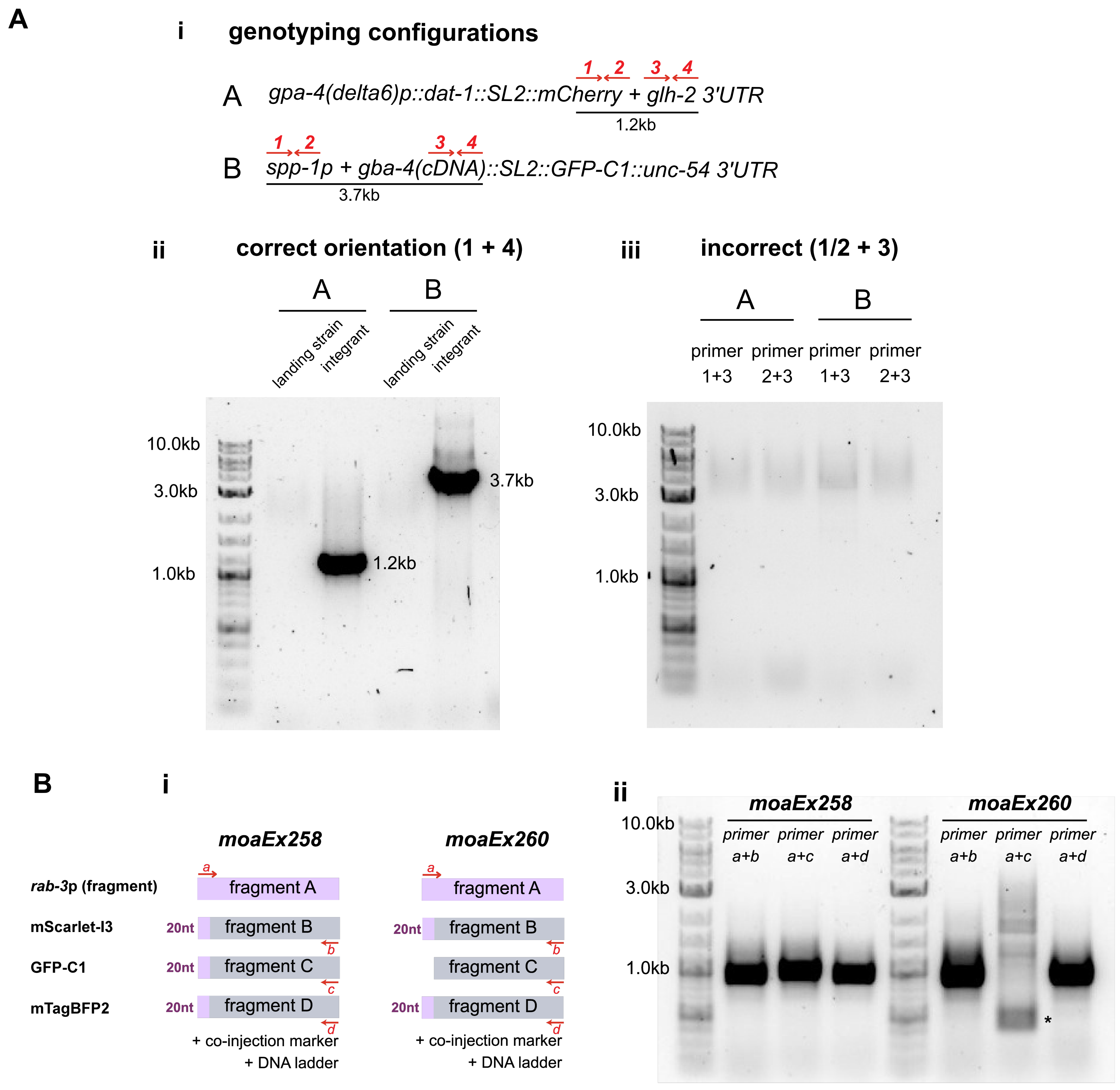

Figure S4. Junction PCR validation of transgenes assembled in vivo.
(A) Schematic overview of genotyping strategy used to verify correct assembly and orientation of integrated transgenes. (i) Primer positions and expected amplicon sizes for two representative transgenes: A, *gpa-4(delta6)*p*::dat-1::SL2::mCherry* + *glh-2* 3′UTR; B, *spp-1*p + *gba-4(cDNA)::SL2::GFP-C1::unc-54* 3′UTR. Red numbers indicate primer binding sites used for junction PCR. '+' indicates position of homology between PCR products. (ii) PCR products generated using primer pair 1+4 confirmed correct full-length assembly in the expected orientation for both constructs. Bands were detected at the predicted sizes (1.2 kb and 3.7 kb, respectively). (iii) PCR using reverse-orientation primer combinations (1+3 and 2+3) did not produce the expected products, supporting correct insertion orientation. (B) Junction PCR analysis of transgenes assembled from three pairs of overlapping DNA fragments in vivo. (i) Schematic representation of fragment design for independent extrachromosomal arrays moaEx258 and moaEx260, showing overlapping 20-nt homology regions between adjacent fragments (A–D), where present. (ii) PCR across each assembly junction (a+b, a+c, and a+d) detected products of the expected sizes in both arrays, consistent with homology-dependent but not homology-independent assembly. '*' indicates non-specific PCR band.
