# Supplementary material for: FLInt 2.0: robust and customizable single-shot integration in C. elegans
Source: G3 (Bethesda). 2026 May 28;16(8):jkag138. doi: 10.1093/g3journal/jkag138 (PMC13439921; doi:10.1093/g3journal/jkag138)
Supplement: Supplementary file 1 [file jkag138_supplementary_data.zip › Figure_S5_G3-2026-406865.pdf]

Figure S5

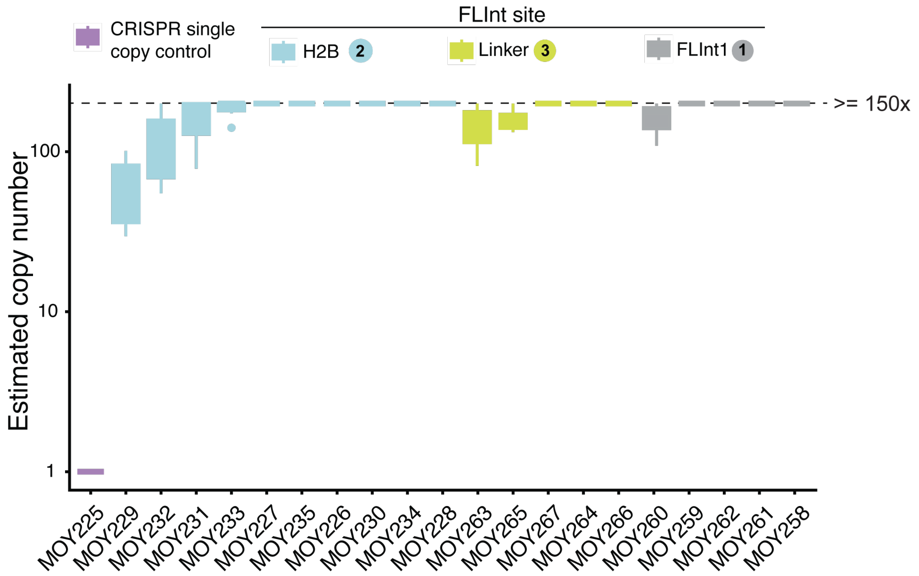

Figure S5. Copy number estimates of integrated transgenes generated via FLInt
qPCR-based copy number estimates of the indicated transgenic strains generated via the indicated integration methods. Boxplots represent estimated copy number of 3 independent biological replicates derived from 3 technical replicates each. Color indicates integration method. Dashed line indicates upper limit for accurate copy number estimation determined using a standard curve, and data are bounded at this level.
