# Supplementary material for: FLInt 2.0: robust and customizable single-shot integration in C. elegans
Source: G3 (Bethesda). 2026 May 28;16(8):jkag138. doi: 10.1093/g3journal/jkag138 (PMC13439921; doi:10.1093/g3journal/jkag138)
Supplement: Supplementary file 1 [file jkag138_supplementary_data.zip › Supplementary_Tables_and_Files_G3-2026-406865/Supplemental_Files/File S1 - tdTomato landing sequence.docx]

**tdTomato landing sequence (miniMos insert)**

***eft-3p + spacer***

gcacctttggtcttttattgtcaacttccattggttcttccattgtttctgttaaattaatgaatttttcataaaataaagacattatacaatataaaaatgaagaatttattgaaaataaactgccagagagaaaaagtatgcaacactcccgccgagagtgtttgaaatggtgtacggtacattttcgtgctaggagttagatgtgcaggcagcaacgagagggggagagatttttttgggccttgtgaaattaacgtgagttttctggtcatctgactaatcatgttggttttttgttggtttattttgtttttatctttgtttttatccagattaggaaatttaaattttatgaatttataatgaggtcaaacattcagtcccagcgtttttcctgttctcactgtttagtcgaatttttattttaggctttcaacaaatgttctaactgtcttatttgtgacctcactttttatatttttttaatttttaaaaatattagaagtttctaggataattttttcgacttttattctctctaccgtccgcactcttcttacttttaaattaaattgtttttttttcagttgggaaacactttgctcacaagtttgtacaaaaaagcaggctcaaaa

***tdTomato and linkers***

atggtgagcaagggcgaggaggtcatcaaagagttcatgcgcttcaaggtgcgcatggagggctccatgaacggccacgagttcgagatcgagggcgagggcgagggccgcccctacgagggcacccagaccgccaagctgaaggtgaccaagggcggccccctgcccttcgcctgggacatcctgtccccccagttcatgtacggctccaaggcgtacgtgaagcaccccgccgacatccccgattacaagaagctgtccttccccgagggcttcaagtgggagcgcgtgatgaacttcgaggacggcggtctggtgaccgtgacccaggactcctccctgcaggacggcacgctgatctacaaggtgaagatgcgcggcaccaacttcccccccgacggccccgtaatgcagaagaagaccatgggctgggaggcctccaccgagcgcctgtacccccgcgacggcgtgctgaagggcgagatccaccaggccctgaagctgaaggacggcggccactacctggtggagttcaagaccatctacatggccaagaagcccgtgcaactgcccggctactactacgtggacaccaagctggacatcacctcccacaacgaggactacaccatcgtggaacagtacgagcgctccgagggccgccaccacctgttcctggggcatggcaccggcagcaccggcagcggcagctccggcaccgcctcctccgaggacaacaacatggccgtcatcaaagagttcatgcgcttcaaggtgcgcatggagggctccatgaacggccacgagttcgagatcgagggcgagggcgagggccgcccctacgagggcacccagaccgccaagctgaaggtgaccaagggcggccccctgcccttcgcctgggacatcctgtccccccagttcatgtacggctccaaggcgtacgtgaagcaccccgccgacatccccgattacaagaagctgtccttccccgagggcttcaagtgggagcgcgtgatgaacttcgaggacggcggtctggtgaccgtgacccaggactcctccctgcaggacggcacgctgatctacaaggtgaagatgcgcggcaccaacttcccccccgacggccccgtaatgcagaagaagaccatgggctgggaggcctccaccgagcgcctgtacccccgcgacggcgtgctgaagggcgagatccaccaggccctgaagctgaaggacggcggccactacctggtggagttcaagaccatctacatggccaagaagcccgtgcaactgcccggctactactacgtggacaccaagctggacatcacctcccacaacgaggactacaccatcgtggaacagtacgagcgctccgagggccgccaccacctgttcctgtacggcatggacgagctgtacaagtacccagctttcttgtacaaagtggagggatccgcccgggcaactagt

***H2B + spacer***

ccaccaaagccatctgccaagggagccaagaaggccgccaagaccgtcgttgccaagccaaaggacggaaagaagagacgtcatgcccgcaaggaatcgtactccgtctacatctaccgtgttctcaagcaagttcacccagacaccggagtctcctccaaggccatgtctatcatgaactccttcgtcaacgatgtattcgaacgcatcgcttcggaagcttcccgtcttgctcattacaacaaacgctcaacgatctcatcccgcgaaattcaaaccgctgtccgtttgattctcccaggagaacttgccaagcacgccgtgtctgagggaaccaaggccgtcaccaagtacacttccagcaagtaaactagttagcattcgtagaattccaactgagcgccggtcgctaccattaccaacttgtctggtgtcaaaaataataggggccgctgtcatcagagtaagtttaaactgagttctactaactaacgagtaatatttaaattttcag

***unc-54 3'UTR + spacer***

catctcgcgcccgtgcctctgacttctaagtccaattactcttcaacatccctacatgctctttctccctgtgctcccaccccctatttttgttattatcaaaaaacttctcttaatttctttgttttttagcttcttttaagtcacctctaacaatgaaattgtgtagattcaaaaatagaattaattcgtaataaaaagtcgaaaaaaattgtgctccctccccccattaataataattctatcccaaaatctacacaatgttctgtgtacacttcttatgttttttacttctgataaatttttttgaaacatcatagaaaaaaccgcacacaaaataccttatcatatgttacgtttcagtttatgaccgcaatttttatttcttcgcacgtctgggcctctcatgacgtcaaatcatgctcatcgtgaaaaagttttggagtatttttggaatttttcaatcaagtgaaagtttatgaaattaattttcctgcttttgctttttggggtttcccctattgtttgtcaagatttcgaggacggcgtttttcttgctaaaatcacaagtattgatgagcacgatgcaagaaagatcggaagaaggtttgggtttgaggctcagtggaaggtgagtagaagttgataatttgaaagtggagtagtgtctatggggtttttgccttaaatgacagaatacattcccaatataccaaacataactgtttccaactttattatacatagttgataattcactggccggatgggccatggcac

***Cbr unc-119(+)***

gcccttctattacagggtttctgataacaaaccggttattaactcccaacaagggatgatttcaattcatcatgctcaaattgacccaaattaagttacatgacaaattcatcgcccttttcaactcttcttggctcatcatctgttattctgttcctattctgtctgcaccctatacccctttgcatactctctcgtcattcctttcttggatagtgtcttcttctccccagctctgctacttctatgacttgcgcccgctgcttttccgctcgttcctctcctctctgacgtcttcttctgcttcttttctccttcctttttcccttctcaatcttcctctttttcccatttctgtcaatcattcgaagaagaagaagaagaagaccctcattcatttcatttttttttctgtcggtgtgtgctgctcggttaagagtgagctctctattccacgtcttcttctttttcttcttcgattcgaatcaatcactccaccaaaacgcattcgtttttgggattcaccccgcggttcgcaataggtttctttttcaaattattagcgttataaatagaaaaatggtggagtttcaataaaaatgataatttacaaagtgtattttgattacatgtactcaaaaaggttgaaatttttcataccagttttccggaaatccatctgatatcattatcgtatttttctttttaaaaatgttttcaaaaaaaaacaaaatatagctggttatttggcaccctctaattaccattttcctgtcacaccacactcttttccttcttccacttctttccgttttcagccgcttccaaccaaaccgatatgaaagccgagcaacaacgatcgattccacccggctcggcgaccttcccgtcgcaggtgagactcagaaaactagagaaaccgctcaactaactcttgatatccgatttcattcttttctcttttctttttgttgaacttttccatatttccagatgccacggccaccaccaagcaccgaacaaggaatcacaacggaatcggagcttgcgaagaaagctcaaatcactccgaacgacgttttagcacttccgggaatcactcaaggtatcttgctccctttggatttcagtttaaaacataaatttaggattcttatgctccccatctgcgaacatctataacatcgagttcaccaagttccaaatccgtgatctggacactgagcaagtgcttttcgagatcgccaaaccggagaacgatcaggagaatgatgagtcgccacaggagtcggcaagatacgtgcgttatagatttgctccaaactttttgaaactcaaaacggtcggagcaactgtggaattcaaagtaggagacatcccaatccatcatttccgaatgatcgaacgtcacttcttcaaagatcgccttctgaagtgttttgactttgaattcggattctgtattccgaattcacgaaacaactgtgaacatatctatgagttccctcaactctctcaacaactcagtgagtcattattctaaaaagtacaattcaaaagactaatctctttttcagtggacgacatgatcaacaatccaaacgagactcgttctgacagcttctatttcgtcgataacaaactcgtcatgcacaacaaagccgactactcatatgatgcataaatatttaatacaaaaatgttctggataattattctgtcgaatagaaaaaaaactccaaatgtgattaaattccaataattcctgtctagtttgttccttccccttccccttctcatgttcaatgcattcctaagcttttcagttccccccttgtttctatattttttcgctgtcctgtcacactcgctaaaaacactaatcacacggaaatctgttttcaataaaaactccaactttaactcattttcaatttcaactgaaagattttttcattagagaatgtc
