# Supplementary material for: FLInt 2.0: robust and customizable single-shot integration in C. elegans
Source: G3 (Bethesda). 2026 May 28;16(8):jkag138. doi: 10.1093/g3journal/jkag138 (PMC13439921; doi:10.1093/g3journal/jkag138)
Supplement: Supplementary file 1 [file jkag138_supplementary_data.zip › Supplementary_Tables_and_Files_G3-2026-406865/Supplemental_Files/File S2 - PCR products for injections.docx]

***gpa-4(delta6)*p::dat-1::SL2::mCherry / *glh-2* 3’UTR combination**

**fragment 1: *gpa-4(delta6)*p::dat-1::SL2::mCherry (6399bp)**

cgatgatcattggaaatgcggtttcccgttttcaaagatttttcatcagttttcaattgtttttgttgttgattttgaaattgctgcctattctgttctgatttttcgaaaactaaaaatctttgaaaaatgaatgtaaatttataattctattttcgaccataaaattcagattctctaaatataatcaccgtcatcatgggataactacaatctgacagaagacagagactcgagaaaatgtgaatacaaattggctgatatagattttttcagaagcaagtagtcagtttctttgattttaatattatgatacccaattttgcgactttcgatacgtaggtctctaaacgatcgaaattttacaactacagtacccccgaatttttttttgcgatttttgttttccaaaaattcaaagtccaagttctacaaatgttgaaatattaagcaattttggtgcaagccaggaatgggcggcaactgtcgttcggcaaattcgataatatttgccggaaattttccaattccgagaaattgccggtttgccgatttgccggaaatttttgttttcggcaaattgctgatttgccgtttgtcggatatcagatttgtcagaaacgttgagagcgattgtttaaagacggaaactcttaaactgtaccttttaaaaatttttcatgttttctttagatatttcatataatttctacacttttcaaaatagatgtaggaacattcataggatgcgtacaattttgccggttaaaattgaagttctgaaattacttttaaaaaatgtacatattttcggcaatttccggtttccgacaaatcggcaatttatcggtttgccgaaatttttcaattccggcgatttgccgctttgtcgatttgccggaaatttcaattccggcaatttgccggaaatttcaattccggaaatctgccgctttgtcaatttgccggaaatttcaatcccggcaatttgccgatttgccggaaacttcaattccggcaatttgccgatttgccggaaacttcaattccggcagtttgccgatttgccggaaaaaattctacattttttatattgcaattttgtttgaattattcgtgttaaaacattgtaggagtcgaaacatacgacattgctttgtatttcctcaaaagctcatatgtttcaaaattttgtaaatttgccaaaacttttagatttttcaacatttccggtcaaagtttttggagtgttttattttaatattcggtcgttttggatctttataagtgtatttaggcaaaatctctacagacgctacttccacctttaattattgaaacaattcaaaagtgtcccaaaaaatcaccgtccaaatgcctgcgtctctgcgcggagacacatcacaattcaaatgatgtttgttttttttttggcgccaaaacttctagaactttttctcctctctcttttgttattgattagtctttctgatacgtgttgtactgaaaaggttgggaaacttctggaacatccattaccttgtggcaactaaagggaaatagttatttaaaacaggaaggtgatattgagaaggtgtttgtttattctttatttttaaataaaaaaaaagaatcggggataaattgaaaaaaaatcacaaaaactaattatcagaagactaattatgccacgtggtgttttgcgaagtgttagcctagaaactgttccctttattcgtccttctcattactctaaattaatttccctcatggttttttaagggtagagccacttattcattttgtgaacacttttcaacaatgcagttggtgcctacaggtatttcgttttttgcccgactcttatttatctgtagactttcgttataaaacaaagctatctttctgaaattgttattacgtagacgcctcaagatgagaatcttcgccaaatcttacgcacatgggtctcgccacgcaaacaaagaatatgatacaatagcggtctgcaaattgacatgagttacagtaattcgttctccgcgtggcgagacccgcggctaaagttctcatccttagagaccaacgtaacactctgggtatagtataacttaccattaaaatttttttttaagttttataattcttcacagggtttaacattagattaaaaattagtcggtgacttctaactaaagttaaaatcaaatcttcagacgatcccgacgaaaaaatcggtcggacgtctaatggcatgcaaaatgcaactcttcctattgatggaccagttaatacagaacccaaagatccagcaagagaacagtggtctggaaaggtaagatccatcttgcctgtaattctacatatgaacccatcttttcagctggacttccttctctcagttgtcgggtttgctgtagatttgggaaatatatggcgatttccatatctttgcttcaaaaatggaggaggagtatttctgattccttattctataatggtcctgttgacaggagttccactattctacatggagttgtgcctcggccagtattatagaaaaggagcaatcacaacttggggaagaatatgtccgttgttcaaaggaatcggatattgtgttattttaaccgcattctatgtggatttcttttataatgtgatccttgcctgggggcttcattatttatatacttcattcaggtgaggacaaaatatgcttattttaggtattataaagacatagtaaaaccaaataatttttaaatcttataaatttttaccgccataatttggaaaactattgcaattaatgtcaaaaatcttgtctaagtctaccaaaaatgactaaagttctacaaagcctctggtaaaatttcaagaaaaaaattacagtttttttaaaacttgatactaattatttttagatatctattcggatatcttgccaatgctataccagaaaaattttaaaaaccaccattccttttaattttgccatattcactttattttcagttttaacctgccatgggcatcctgtaacaacagttataactctcctgcttgttacgaaccacactggtcagaagacggaacagcaatgtgtcgaagtgcaaatcaatctgtctcagctgaaaagatttcagctgctgaagaatacttttagtaatttctaaacttttttgataatttctaaaaaaaatatatatgtttcagtaagggatttctggggctccatgaagcaaatgcaccgaactctcacgttattcgaagtgtcaccgatctgggaaatgtacgttgggacattgctgtaagtatacaaaaattaatgaatgaaatttgaaaattctaatttaaagctttccctcttcgttgtgtatctcatttgctacttttcaatgtggaaaggaatccatacttctggaaaagttgtctggtttactgctctatttccatatgttgtactgggaattctattcattcgtggagtgactctacccggatggcaaaacggaatcgaatattatcttcgacccaactttgaaatgctcaaggtttcgagtttcagatagaaaaattcaaatttcaagctttgtagagaccatcggtctggcaagatgctgccacgcaagtatttttctcattagggccaggattcggagttctcatggcatactcgtcatataatgatttccataataatgtatatgtgtaagttttggaatttttgaattacaatttttttgaaatttttacagggatgctcttttcacatcattcatcaattgtgcaacatcatttctctcagggtttgtgattttctccgtactcggctacatgtcctgcaaatctggaaaaccaattgaagcagttgctcaagaaggccctggactagtatttgtagtctatccagaagcactctcaacaatgccatatgctccattctggtctgtgctctttttcttaatgctcatgacacttggccttgattcttcagtaagtctgaatcgtaagaaaacctatagcagattattttagttcggaggatctgaagctatcatcaccggcctttcagatgaatttccaatattgaaaaagaacagagaagtgttcgttggttgtttgtttgctttttacatggtaattggaattgctatgtgtacagaggtaaccttttcaccgaaaactatcttcatcaaaaatttttgaagggtggaattctaatcatggaatggctcatcatctatggaactacatggggcttattgattgcagtgttctgtgaagcaatggtcattgcatacatctacggtaggtgtgctctgaatctcaaaatttccaaaatttcacaggtctgcgacaatttgttcatgacgtcaaagagatgatgggattccgcccgggaaattattggaagttttgctggagctgtgccgcaccattcattttattggtaagagtctggtttttggcgccaactgcgttggcccaaacttttcaagtgtcgttttgaatttgcaccagaacttgacaaaaaatggatcacacttgattcaaacttggcgcttttaacaacatttgcactcacaaactttttattaaaaaataaattttacatcttttccaataggaaggtgtggcaattttctatatgtagcggaaaaagtatttacaacatcatccaacatgaccaaattaccaatattccagtcgatgatcacttccaacttcatcaattatcaagccttgacctaccaggactacacatacccaaccgcggcaaacgttataggaattatttttgcgctctcaggcgcctcatttattccattggtaggaatctacaaattcgtcaatgcgagggggaacacgatatctgaggtcagtgattaaaacaataattgggttgagatcaggttcgaactgtattagtcatatacctaaaatctgtgaattcgatttcggaatatgccatatgttataagttggattttttaaaatttattggtaatgtcaaccattccgagcagaaatcagtctcgaaagcgtaggatagaatgaaatagttagatatgaagcactgttttggtcttactttctggcaaaattttgcaactctctcccagaattttgaaaatttctgcaaaaaaaatttcagcgtactgacaaagctgtctaccaaatttctgcaacgttttgcaactaaactccaaaacaaagttgacaacgaacaccgttctctcaattggaacattttaatactaaaatatattaactaatacatttcaaacacacttaaagcttgtgtatttttttttcagaaatggcaacgagtcacaatgccttatcgaaaaaggccgaatcaaacagaatatattccaattccaaccactcaaccgcactctgacataatgctatgaggtaccgctgtctcatcctactttcacctagttaactgcttgtcttaaaatctatgcttctctttagtatctaaaattttcctagaagcttacaagtatataaatggtctcttctcaataaaggttgtatatttattcatcttattgaatctgccatttcctcgtttttgcgagtttatataccttccaattttctttctattgtattttcaacttctaattttaattcagggaaactgctgtaccggtagaaaaaatgctcgagatggtgagcaagggcgaggaggataacatggccatcatcaaggagttcatgcgcttcaaggtgcacatggagggctccgtgaacggccacgagttcgagatcgagggcgagggcgagggccgcccctacgagggcacccagaccgccaagctgaaggtgaccaagggtggccccctgcccttcgcctgggacatcctgtcccctcagttcatgtacggctccaaggcctacgtgaagcaccccgccgacatccccgactacttgaagctgtccttccccgagggcttcaagtgggagcgcgtgatgaacttcgaggacggcggcgtggtgaccgtgacccaggactcctccctgcaggacggcgagttcatctacaaggtgaagctgcgcggcaccaacttcccctccgacggccccgtaatgcagaagaagaccatgggctgggaggcctcctccgagcggatgtaccccgaggacggcgccctgaagggcgagatcaagcagaggctgaagctgaaggacggcggccactacgacgctgaggtcaagaccacctacaaggccaagaagcccgtgcagctgcccggcgcctacaacgtcaacatcaagttggacatcacctcccacaacgaggactacaccatcgtggaacagtacgaacgcgccgagggccgccactccaccggcggcatggacgagctgtacaagtaa

**primers for amplification fragment 1 (introducing overlaps)**

- Forward (OLO-01172)
  - attacgccaagcttgcatgccgatgatcattggaaatgcggtttcc
- Reverse (OLO-01212)
  - ttgaaggatatgcagatacatttacttgtacagctcgtccatgccgccggtggag

**fragment 2: glh-2 3’UTR (441bp)**

atgtatctgcatatccttcaaatgttgtccatattgtatcagtaaattataatgccccctttttatatattctccttatcatgtaaatgttctattgattttttgctggtgtaaaacgtttttatagtaaatcctctccacagtgaatttgttaaattgtatcaaaacaagacattgattaaatttgttcgagacttgtggattgtaaaatattttgcagaaaatggcaatattcatttagaaacattacttccataataggaaaaactatttatgagttttcatgtgcctatgcaagttcccactctgtgtgaatttcatcatacaaattgagcaaacataaggtttatgtccagtgtgtgttctgaaataatgaaaaatagtcttgcaatatgtttctactattcgagaaaaaatgtaattttttttcttatggtaacc

**primers for amplification fragment 1 (introducing overlaps)**

- Forward (OLO-01152)
  - atgtatctgcatatccttcaaatgttgtccat
- Reverse (OLO-00947)
  - accgggttaccataagaaaaaaaattacattttttc

***spp-1*p / *gba-4*(cDNA)::SL2::GFP-C1::*unc-54* 3’UTR combination**

**Fragment 1: *spp-1* promoter (2151bp)**

aaagtaatcgtactattcagagaaaatgagctcgctcaccgacaaaacccgaaatggagccctgaagcggaatctgctccgccaaatgctcaaagcgagcgtcttcaaattcggcgagtttcagctcaagtctgggcagatttcaccgatttatatcgatcttcgcgaatgctttgggcatccggggcttttggtgagttttttaaagtttaaaaaaaaaataatagcttattttaatgatttttatttaaaagaaaactatttccagatgcttatctcagaagcgatatcgaagcaggtggagattagcgaggttcagtacgctggagttcttggaatcccatacgcggctcttccctacgcatcagtcgccgctggaaactatctgaagaagccgctgctcatcgttcgcaaagaggccaagtcctacggcacgaagaagctcatcgagggattgtaccagccaaacgatcgtctgatcctcatcgaagacgtcgtgacgacaggtggaagcatcctggacgtggtaaaagtgcttcacacagagaatttggtcgcctcggatgtattttgcattctggatcgagaacaaggcgggcgtcagaagctccaggacgcgggtgtcacgctgcacagcctgctcgacatgcaaacagttctcacattcttgtactcaactggagcgatcggtgacgagcagtggcacggaattgttcaggttagtcaaattatctcgattttctatttaaaaataaaataattgattatttcaggctctcaaccttccgtacacctccccaacaaagctggaaatcaacagcgagctcgaaaacctgtccagtctaccgtacgtggaaaacgtgcgaactccgcttgcggaacgcgaatccctcacagaatctgcactcatcaagaaaattcttggaatcatgcgccggaagaagtcgaatctgtgtctcgctgtcgactacactaccgtcgagcagtgccttcagatgatcgagctcgctggtccatttgtgctggcgatcaagcttcacgccgacgctatcaccgatttcaacgaggaattcacgaggaagctgacgacgatggcaaatgatatggactttatcattttcgaggatcggaagtttggcgacactggaaacacgaaccttcttcagctcactggagcccagaaaatcgccaactgggcggatgttgtgactgttcatgccgtacaaggaagtgacagcattgcaggagtcttcaggaagctcgccaaagatccgacttacaggctcagtggagttctcctcatcgcgcagctcagcaccaaaggatctttgactgcgctggaaggatacaccgagaccgccgtcaagattgccaacgagaatcgtgacgtgatttctggattcatcactcagacgagagtgtctgcttgctcggatcttctcaattggacgcccggtgtgaatctcgacgcgaaatccgactcggccggtcagcagtggagaggtgtcgacgaggcgatcgaagtacagcagaacgacatcatcatcgtcggaagaggcgtcacgtcgtccagtgagcccgttcagcagctcaagaggtttgtttttttttttttaattaaatttttaaatgtcaattttctctttcagatatcgacaaatcgcctgggatgcgctcacgagaagcgacgatagcatttgaaagttgaaaaatccggagagcctatttgtaaagaagaaatgtgaatatttatctaattacgtatttatttaactttgttgattttgttcaagcttccccagaattgttgaaataagtaaattgattgcctccatcttcctcaaattatcaagaaatcacatgcccacaccatttactttactgctaacctgattaatcaatattttctccgtcaaggcgaaaagctactcctctaagcgatcttatcagttcgaaaaatcggcatctgatgtgtgaacgacccggtgtgtattctataatgtcctttgatcttttcctccatgctgaaaacatcagaaacgattcgattccttatcagtgccgccgcgaagttgataaaagacacgagcatcactgctccgggtgcattttcgcttcatttctccagtttttcatcttaaaatctacaga

**primers for amplification fragment 1 (introducing overlaps)**

- Forward (OLO-00901)
  - attacgccaagcttgcatgcaaagtaatcgtactattcagagaaaatgagctcgctc
- Reverse (OLO-01091)
  - tctgtagattttaagatgaaaaactggag

***Fragment 2: gba-4(cDNA)::SL2::GFP-C1 ::unc-54 3’UTR (3483bp)***

atgattttgaatatatccgtttctttgctaatatttctggcgttttatggattttcaagcgatgcaaagtcccttccttgctccgaagtgaagaaagagtatggaatagtttgtcgttgcaatgccacgtattgtgatacaattgaaccacttggaactgttacttctgggaaagcagttgtctatacaacatcaagaaatggaaaaagaatgaacagaagtgaattgaagcatacaacttcttctacagccaaaacaaaagtatatgtgaacacaactcaaagttttcaaccggtaatgggatttggagctgcattcactgatgctgctggaattaatatgaagatgctgccacagacaatgcaagatcaaattattcagcaatacttttccgatgatggtctcgggtatgtttttggacgagttccaatggcttcaactgacttttcaactcacgagtacagttatgacgacgttaaatttgattttgatctcaaaaatttcaatttaacagtggaagatctccagtataaaattccatttatcaagaaggcaatgacagcttctggagggaaattaaagctatttgcaactccatggagctccccaggatggatgaaaacttctggaagaatggttggtgccggagagctattgggagatcagaatggaaaatactatcagacatgggcacagtactttgtaaaattcttcgaagcctatcacgctcaaggtattgacttttggtctttgacaccacaaaacgagccaacaactggaattgatccactttggaaatggcaaacactctttttcgatgcatcaatggaacgaaattttataaagaaacttttgggaccagcacttgcttcatcaccagtgactaagaatttgaagattatgattaatgatgatcagagaattaatttgccacactggccaaatgtaattttaacggatccaactgctgctcaatacgtccacggaattgcaattcattggtatgaagacttcattgacccagcaaccgtactcactgaaactcacgagaagttcccagactatttccttttggcaactgaagcatgtgctggatacttcccagcagatggaccaaaattgggatcatggtctagagctgaacaatatgcaaatgatttgattaaagatatgggaaattgggttggaggatgggttgattggaattatattcttgatttacaaggaggaccaaatcttgccaaaaactttgtcgattcaacaataatcgtgaatgcaactgctcaagagtactacaaacaaccaatttggcatgtaatggctcaattcagtaaatttgtgaaaccgggagctattcgtgttggaataaacattattgagaaatcagttgatgttgagggtctttcatttttgaatcaagatggtaccaaaactgtggttttattgaataaaaatgaggtactcagcttcgacgtggcaatcagtgacgtcagtgctccaaatgtgatctatgatttgacaatccaaccgaattcactgattacaattgtttataaataagctgtctcatcctactttcacctagttaactgcttgtcttaaaatctatgcttctctttagtatctaaaattttcctagaagcttacaagtatataaatggtctcttctcaataaaggttgtatatttattcatcttattgaatctgccatttcctcgtttttgcgagtttatataccttccaattttctttctattgtattttcaacttctaattttaattcagggaaactgctgtaccggtagaaaaaatgctcgagatgagtaaaggagaagaattgttcactggagttgtcccaatcctcgtcgagctcgacggagacgtcaacggacacaagttctccgtctccggagagggagagggagacgccacctacggaaagctcaccctcaagttcatctgcaccaccggaaagctcccagtcccatggccaaccctcgtcaccaccttctgctacggagtccaatgcttctcccgttacccagaccacatgaagcgtcacgacttcttcaagtccgccatgccagagggatacgtccaagagcgtaccatcttcttcaaggtaagtttaaacatatatatactaactactgattatttaaattttcaggacgacggaaactacaagacccgtgccgaggtcaagttcgagggagacaccctcgtcaaccgtatcgagctcaaggtaagtttaaacagttcggtactaactaaccatacatatttaaattttcagggaatcgacttcaaggaggacggaaacatcctcggacacaagctcgagtacaactacaactcccacaacgtctacatcatggccgacaagcaaaagaacggaatcaaggtcaacttcaaggtaagtttaaacatgattttactaactaactaatctgatttaaattttcagatccgtcacaacatcgaggacggatccgtccaactcgccgaccactaccaacaaaacaccccaatcggagacggaccagtcctcctcccagacaaccactacctctccacccaatccgccctctccaaggacccaaacgagaagcgtgaccacatggtcctcctcgagttcgtcaccgccgccggaatcacccacggaatggacgagctctacaagtaatcgctacccagctttcttgtacaaagtggtgatatctgagctccgcatcggccgctgtcatcagatcgccatctcgcgcccgtgcctctgacttctaagtccaattactcttcaacatccctacatgctctttctccctgtgctcccaccccctatttttgttattatcaaaaaacttctcttaatttctttgttttttagcttcttttaagtcacctctaacaatgaaattgtgtagattcaaaaatagaattaattcgtaataaaaagtcgaaaaaaattgtgctccctccccccattaataataattctatcccaaaatctacacaatgttctgtgtacacttcttatgttttttacttctgataaatttttttgaaacatcatagaaaaaaccgcacacaaaataccttatcatatgttacgtttcagtttatgaccgcaatttttatttcttcgcacgtctgggcctctcatgacgtcaaatcatgctcatcgtgaaaaagttttggagtatttttggaatttttcaatcaagtgaaagtttatgaaattaattttcctgcttttgctttttggggtttcccctattgtttgtcaagatttcgaggacggcgtttttcttgctaaaatcacaagtattgatgagcacgatgcaagaaagatcggaagaaggtttgggtttgaggctcagtggaaggtgagtagaagttgataatttgaaattggagtagtgtctatggggtttttgccttaaatgacagaatacattcccaatataccaaacataactgttt

**primers for amplification fragment 2 (introducing overlaps)**

- Forward (OLO-01227)
  - ttcatcttaaaatctacagaatgattttgaatatatccgtttctttgctaatatttctgg
- Reverse (OLO-01252)
  - cccgtacggccgactagtagaaacagttatgtttggtatattgggaatgtattc
